# Coilin and SUMOylation influence PARP1 dynamics and the DNA damage response

**DOI:** 10.1242/jcs.263953

**Published:** 2025-05-21

**Authors:** Sara K. Tucker, Blaise C. Seale, David T. Brown, Michael D. Hebert

**Affiliations:** Department of Cell and Molecular Biology, The University of Mississippi Medical Center, Jackson, MS 39216-4505, USA

**Keywords:** Coilin, SUMOylation, PIAS4, PARP1, DNA damage response

## Abstract

Coilin is a nucleoplasmic protein that is enriched in some cell types in the Cajal body (CB). CBs take part in the biogenesis of many different types of ribonucleoproteins (RNPs), such as small nuclear RNPs. Coilin is known as the CB marker protein and is required for CB formation. The function of nucleoplasmic coilin is less understood and has been shown to impact protein modification by SUMO, the small ubiquitin-like modifier. Additionally, it is known that coilin is recruited to sites of DNA damage caused by UVA exposure or expression of herpes simplex viral protein. PARP1, a DNA damage response protein, has been shown to be SUMOylated by PIAS4, a SUMO E3 ligase that associates with coilin. Here, we show that SUMOylation of PARP1 is lessened when coilin is suppressed. We also found that coilin knockdown and a SUMO inhibitor drug, TAK-981, influence the dynamics of PARP1 in response to micro-irradiation. Additionally, we find that the SUMOylation status of coilin influences its mobility in the CB and recruitment to sites of DNA damage. These data demonstrate that coilin and SUMOylation both have an influence on the DNA damage response.

## Teaser

**Summary:** The involvement of coilin in protein SUMOylation is not fully understood. We have found coilin influences the SUMOylation and dynamics of PARP1, a DNA damage response protein.

## INTRODUCTION

Coilin is a protein that is ubiquitously expressed in the nucleus of all human cell types. In some cell types, such as neuronal and cancer cells, coilin is also enriched in a subnuclear domain known as the Cajal body (CB) (Andrade et al., 1991; Young et al., 2000). CBs play an important role in the biogenesis of many types of ribonucleoproteins (RNPs), such as small nuclear RNPs, which are involved in splicing and telomerase (Kiss, 2004). Coilin is required for CB formation and contains a large central region of intrinsic disorder that, along with the structured N- and C-termini, provide an interaction surface for proteins and small RNAs enriched in the CB (Arias Escayola et al., 2025; Machyna et al., 2014). Besides coilin, other proteins enriched in the CB include the survival of motor neuron protein (SMN; also known as SMN1), which is mutated in most cases of the neurodegenerative disease spinal muscular atrophy, and WRAP53, which plays a role in the telomerase holoenzyme assembly (Liu and Dreyfuss, 1996; Mahmoudi et al., 2010; Matera and Frey, 1998).

Although coilin has been well characterized regarding its role in CB formation and composition, relatively little is known about the function of nucleoplasmic coilin despite the fact the many cell types lack CBs yet still express coilin and, in cells with CBs, the majority of coilin is nucleoplasmic (Lam et al., 2002). One possible function for nucleoplasmic coilin is in the DNA damage response (DDR), as evidenced by the recruitment of coilin to sites of DNA damage caused by UVA irradiation (Bártová et al., 2014) or herpes simplex virus type1 infected cell protein 0 (Morency et al., 2007). The effects of coilin at sites of DNA damage and the mechanisms by which coilin is recruited to these sites are largely unknown. Another function of nucleoplasmic coilin is the promotion of protein modification by the small ubiquitin-like modifier (SUMO) (Lett et al., 2023). Protein modification by SUMO involves the covalent attachment of a SUMO molecule to a target lysine residue through a SUMO-specific activating enzyme (E1), a conjugating enzyme (E2, Ubc9) and a ligation enzyme (E3) (Mahajan et al., 1997; Wang and Dasso, 2009). *In vitro*, the E1 and E2 enzymes are sufficient to SUMOylate target proteins; however, E3 ligases increase the specificity and efficiency of target protein SUMOylation (Pichler et al., 2017). SUMOylation has been shown to influence numerous cellular processes including protein stability, protein localization and the DDR (Sarangi and Zhao, 2015; Wilkinson and Henley, 2010). Although there is only one E2-conjugating enzyme, Ubc9, there are numerous E3 ligases and SUMOylation occurs in groups that are functionally and physically linked (Psakhye and Jentsch, 2016). In addition, SUMO–protein interactions can take place via a SUMO-interacting motif (SIM), thus creating a multitude of possible interactions between proteins facilitated by SUMOylated residues and SIMs (Minty et al., 2000). SUMOylation is thought to provide the ‘glue’ that holds these specific SUMO protein groups together and facilitates their biomolecular condensation (Gutierrez-Morton and Wang, 2024). Indeed, coilin is a highly SUMOylated protein and has a large region of intrinsic disorder that both likely contribute to the condensation and liquid–liquid phase separation of the CB (Tucker et al., 2024). Given the presence of SUMOylated residues, SIMs and intrinsic disorder in coilin, and the interaction of coilin with SUMO, Ubc9 (Lett et al., 2023) and the SUMO E3 ligase PIAS4 (Sun et al., 2005), it is possible that coilin also promotes the SUMOylation of proteins in the nucleoplasm (Lett et al., 2023).

Similar to coilin, poly (ADP-ribose) polymerase-1, or PARP1, is a nuclear protein involved in the DDR; however, its role in this response has been much more thoroughly documented (de Murcia and Ménissier de Murcia, 1994). PARP1 enzymatically promotes the post-translational modification ADP-ribosylation (also known as PARylation) to various substrates, including PARP1 itself, at the site of DNA damage (Chambon et al., 1963; Moss and Stanley, 1981; Pieper et al., 1999). This enzymatic activity begins when PARP1 responds to a DNA break and leads to the recruitment of various types of proteins that actively repair the DNA. These repair proteins localize to the site of damage following auto-PARylation of PARP1, which establishes a negative charge on the protein, and thus facilitates its disassociation with DNA to allow for repair proteins to have access (Kang et al., 2022). PARP1 is additionally regulated by other modifications, including SUMOylation (Martin et al., 2009), ubiquitylation (Zhang et al., 2020), and acetylation (Yang et al., 2023). Interestingly, PARP1 has been shown to associate with coilin in both plants and in *Drosophila* (Kanev et al., 2024; Kotova et al., 2009; Spechenkova et al., 2023). Therapeutics known as PARP inhibitors (PARPi) have been used in the treatment of certain cancers to induce lethality by impairing the repair of DNA breaks (Bryant et al., 2005). One of the suggested mechanisms of action of PARPi is PARP1 trapping, leading to an accumulation of breaks. This proposed mechanism suggests that PARPi binds to the enzymatic active site of PARP1 and blocks its ability to auto-PARylate; therefore, ‘trapping’ PARP1 at the site of damage and inhibiting access of repair proteins to the breaks (Farmer et al., 2005). Interestingly, it has been shown that trapped PARP1 is SUMOylated by PIAS4 and that the interference of this SUMOylation might lead to enhanced PARPi sensitivity (Krastev et al., 2022). PIAS4, which has been shown to associate with coilin (Sun et al., 2005), is recruited to sites of DNA damage where it facilitates the SUMOylation of many different substrates (Galanty et al., 2009).

In this work, we set out to investigate the reason for the recruitment of coilin to sites of DNA damage and its role in the DDR. Specifically, we investigated the impact of SUMOylation on the dynamics of PARP1 in the DDR and evaluated the impact of SUMOylation of coilin on its dynamics upon DNA damage. We found that coilin influences both the PARP1 SUMOylation status as well as dynamically altering the PARP1 response to DNA damage. Additionally, coilin dynamics in the DDR is dependent on its own SUMOylation. Collectively, the data presented here demonstrate a mechanistic involvement of nucleoplasmic coilin with the canonical PARP1 DDR, and support a dependence of these dynamics on protein SUMOylation.

## RESULTS

### Coilin positively contributes to PARP1 SUMOylation

**Fig. 1.**
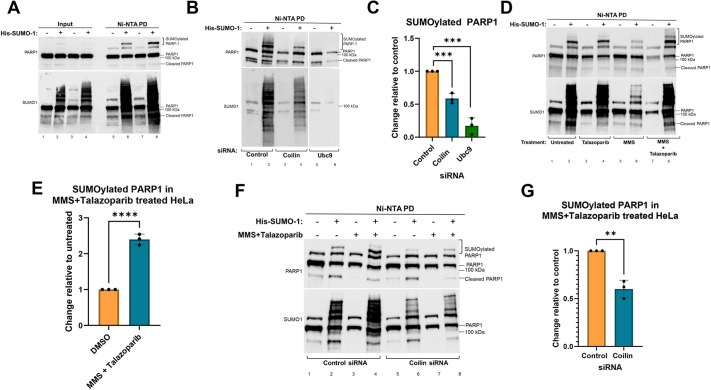
**Coilin is a regulator of PARP1 SUMOylation.** (A) HeLa cells were untransfected (−) or transfected (+) with His–SUMO1 for 24 h and subject to Ni-NTA pulldown (PD), SDS-PAGE, western transfer and probing with anti-PARP1 (upper panel) or anti-SUMO1 (lower panel). Higher migrating SUMOylated PARP1 is indicated (bracket). Blot representative of three experimental repeats. 20 μl of total lysate was used for input. (B) HeLa cells were treated with control, coilin or Ubc9 siRNA for 48 h and untransfected (−) or transfected (+) with His–SUMO1 for an additional 24 h. Ni-NTA pulldown, SDS-PAGE and western transfer was conducted, and the blot was probed with anti-PARP1 (upper panel) and anti-SUMO1 (lower panel) antibodies. Higher migrating SUMOylated PARP1 is indicated (bracket). (C) Quantification of B and other blots. Quantification was done relative to input signal shown in Fig. S1B to control for total lysate loaded in each lane. That was then further normalized to the non-specific binding of PARP1 in the pulldown lanes to control for beads recovered. The amount of SUMOylated PARP1 recovered in control siRNA reactions has been set to 1 and the amount of SUMOylated PARP1 recovered in coilin or Ubc9 KD reactions is shown relative to control. Results are mean±s.e.m., *n*=3. ***P<0.0005 (unpaired two-tailed Student's *t*-test). (D) HeLa cells were transfected with or without His–SUMO1 for 24 h. At the 23 h mark, cells were treated with Talazoparib, MMS or MMS+Talazoparib for 1 h. Lysate was generated and subjected to Ni-NTA pulldown, SDS-PAGE, western blotting, and probing with antibodies to PARP1 (upper panel) or SUMO1 (lower panel). SUMOylated PARP1 is indicated by a bracket. (E) Quantification of D and other blots show a significant increase in SUMOylated PARP1 in cells treated with MMS+Talazoparib compared to untreated, which is set to 1. Results are mean±s.e.m., *n*=3. ****P<0.00005 (unpaired two-tailed Student's *t*-test). Quantification was done as described above. (F) HeLa cells were transfected with either control or coilin siRNA for 72 h. Cells were left untransfected or transfected with His–SUMO1 48 h after siRNA transfection. At 71 h post siRNA transfection, cells were either treated or untreated with MMS+Talazoparib for 1 h. Lysate was collected and subject to Ni-NTA pulldown, SDS-PAGE, western blotting and probing with anti-PARP1 (upper panel) or anti-SUMO1 (lower panel) antibodies. SUMOylated PARP1 is indicated by a bracket. (G) Quantification of F and other blots showing that coilin reduction decreases the SUMOylation of PARP1 in the presence of MMS+Talazoparib treatment. Results are mean±s.e.m., *n*=3. **P<0.005 (unpaired two-tailed Student's *t*-test). Quantification was done as described for C.

To assess whether coilin impacts PARP1 SUMOylation and DDR dynamics, we first verified that PARP1 is SUMOylated using a nickel-nitrilotriacetic acid (Ni-NTA) pulldown approach. For this approach, HeLa cells were transfected with a plasmid expressing His–SUMO1 followed 24 h later by lysate generation and incubation with Ni-NTA beads. In so doing, proteins conjugated to His–SUMO1 are recovered by the Ni-NTA beads, which are subsequently washed and subjected to SDS-PAGE, western blotting and detection of endogenous PARP1 using an anti-PARP1 antibody. As shown in Fig. 1A, a higher migrating species corresponding to SUMOylated PARP1 is detected in the pulldown reactions with His–SUMO1 (top panel, lanes 6 and 8) but not in reactions lacking His–SUMO1 (top panel, lanes 5 and 7). Note that endogenous non-SUMOylated PARP1 is ‘sticky’ and is recovered in pulldowns, with or without His–SUMO1. The non-specific binding of non-SUMOylated proteins in pulldown reactions has also been observed for other proteins, including DGCR8 and small nucleolar RNP components (Lett et al., 2023; Ryu et al., 2021; Tucker et al., 2024). Probing of the same blot with an antibody to SUMO1 shows that SUMOylated proteins are present in higher amounts in the input lanes and pulldown reactions in the presence of His–SUMO1 transfection (Fig. 1A, bottom panel). This same experiment was performed after transfection of His–SUMO2 in place of His–SUMO1, and we found a similar detection of SUMOylated PARP1 when probed with anti-SUMO2/3 (Fig. S1A).

Given that it has been shown that coilin knockdown (KD) decreases the SUMOylation of other proteins including DGCR8, a component of the microRNA biogenesis pathway (Lett et al., 2023), we next examined whether coilin reduction alters PARP1 SUMOylation. For this experiment, cells were transfected with control, coilin or Ubc9 (the SUMO E2 conjugation enzyme) siRNA for 48 h followed by transfection with a His–SUMO1 plasmid and lysate generation 24 h later (resulting in a 72 h KD and 24 h His–SUMO1 expression). Ni-NTA pulldown with the obtained lysate shows that Ubc9 KD, the positive control for reduced SUMOylation, dramatically decreased the amount of PARP1 SUMOylation (Fig. 1B, compare signal in lane 6 to that in lane 2). Likewise, coilin KD also decreased the amount of SUMOylated PARP1, although this reduction is not as profound as that observed for Ubc9 KD (compare lane 4 to lane 2). These data, and other biological repeats of this experiment, were quantified and show a significant decrease in SUMOylated PARP1 with coilin and Ubc9 KD compared to control KD (Fig. 1C). One particular thing to note is the global decrease in SUMOylation seen in both Ubc9 and coilin KD in Fig. 1B. This has previously been noted (Lett et al., 2023) in coilin KD. A typical input for this experiment is shown in Fig. S1B and typical KDs for coilin and Ubc9 are shown in Fig. S1C. PARP1 SUMOylation is also reduced when using a different siRNA to KD coilin, or upon KD of WRAP53, a component of CBs with known roles in the DDR (Bergstrand et al., 2020; Henriksson et al., 2014) (Fig. S1D).

We next wanted to evaluate whether coilin impacts the SUMOylation status of chromatin-trapped PARP1. Previous work has shown that combination treatment with a DNA-damaging agent and a PARPi results in the trapping of PARP1 at the chromatin, where it is unable to PARylate itself or other proteins (Min and Im, 2020) (Krastev et al., 2022). Interestingly, this trapped PARP1 has increased SUMOylation compared to soluble PARP1 (Krastev et al., 2022). To verify that a DNA-damaging agent plus a PARPi increases PARP1 SUMOylation, cells were left untransfected or were transfected with a plasmid encoding His–SUMO1 and then were treated or not with the PARPi Talazoparib alone as a control, the DNA-damaging agent methyl methanesulfonate (MMS) alone as a control, or a combination thereof (MMS+Talazoparib). Lysate was then subjected to Ni-NTA pulldown and, as shown in Fig. 1D, the amount of SUMOylated PARP1 was significantly increased by the MMS+Talazoparib treatment compared to in the untreated conditions (compare the amount of SUMOylated PARP1 in lane 8 compared to that in lane 2, quantification of this and other blots shown in Fig. 1E). Representative inputs for this experiment are shown in Fig. S1E. This experiment was repeated using His–SUMO2, and we found a similar increase in PARP1 SUMOylation upon treatment with MMS+Talazoparib (Fig. S1F). We found that when both recruited and trapped, PARP1 was more highly SUMOylated compared to what was seen for the untreated, only recruited (MMS treatment) or only inhibited (PARPi treatment) conditions. To assess whether coilin promotes the SUMOylation of trapped PARP1, control or coilin KD cells were transfected with His–SUMO1 and then unexposed or not to Talazoparib+MMS. Lysate was then subjected to Ni-NTA pulldown. Coilin KD was found to decrease the amount of SUMOylated PARP1 in the presence of Talazoparib+MMS compared to that obtained in cells treated with control siRNA (Fig. 1F, compare SUMOylated PARP1 signal in lane 8 compared to that in lane 4). Quantification of this and other blots showed that coilin KD significantly decreased the amount of SUMOylated PARP1 with Talazoparib+MMS treatment compared to that seen in control (Fig. 1G). Representative inputs are shown in Fig. S2A. A summary of these findings for SUMOylated PARP1 is shown in Fig. 6G.

### Coilin and the SUMO E3 ligase PIAS4 influence the level of SUMOylated PARP1 in the presence of Talazoparib+MMS

**Fig. 2.**
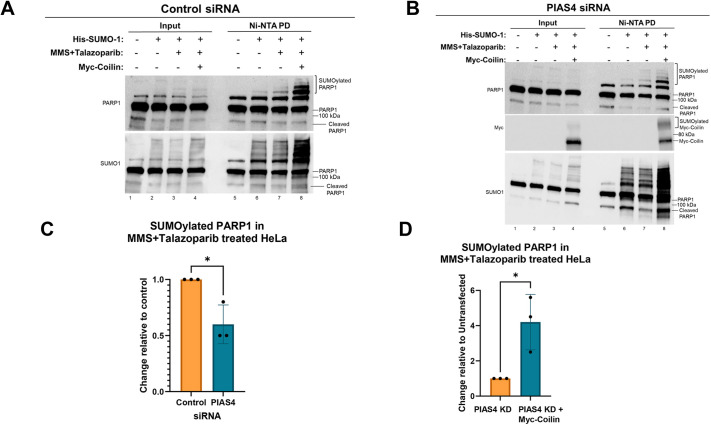
**Coilin and PIAS4 promote the SUMOylation of PARP1 in cells treated with MMS plus Talazoparib.** (A) HeLa cells were transfected with control siRNA 72 h. At the 48 h mark, cells were left untransfected, transfected with His–SUMO1 or co-transfected with His–SUMO1 and Myc–coilin. At the 71 h mark, cells were either untreated or treated with MMS+Talazoparib for 1 h. Lysate was generated and subjected to Ni-NTA pulldown (PD), SDS-PAGE, western blotting and probing with antibodies to PARP1 (upper panel) or SUMO1 (lower panel). SUMOylated PARP1 is indicated by a bracket. (B) HeLa cells were treated as in A, except cells were transfected with PIAS4 siRNA instead of control siRNA. The blot was probed with anti-PARP1 (top panel), anti-Myc (middle panel) and anti-SUMO1 (bottom panel) antibodies. SUMOylated PARP1 and SUMOylated Myc–coilin are indicated by a bracket. 20 µl of total lysate was used for input in A and B. (C) Quantification of A and other blots showing that PIAS4 KD decreases the amount of SUMOylated PARP1 in the presence of MMS+Talazoparib. Results are mean±s.e.m., *n*=3. *P<0.05 (unpaired two-tailed Student's *t*-test). (D) Quantification of B and other blots showing that the expression of Myc–coilin in the presence of PAIS4 KD and MMS+Talazoparib treatment increases the amount of SUMOylated PARP1 compared to reactions lacking Myc–coilin. Quantification was done as described in Fig. 1C, with the amount of SUMOylated PARP1 present in PIAS4 and MMS+Talazoparib set to 1. Results are mean±s.e.m., *n*=3. *P<0.05 (unpaired two-tailed Student's *t*-test).

PIAS4 is a SUMO E3 ligase that has been shown to interact with and promote the SUMOylation of PARP1 (Martin et al., 2009). Additionally, PIAS4 has been shown to associate with coilin (Sun et al., 2005). To examine whether coilin and PIAS4 together contribute to the SUMOylation of PARP1, we first monitored whether PIAS4 KD altered PARP1 SUMOylation. In agreement with a previous report (Martin et al., 2009), there was less SUMOylated PARP1 when PIAS4 was reduced compared to that seen in controls (compare the PARP1 SUMO signal in Fig. 2A, lane 6 to the signal in Fig. 2B lane 6). We also noted that SUMOylated PARP1 was decreased upon PIAS4 KD in the presence of Talazoparib+MMS compared to that seen in control KD (compare the PARP1 SUMO signal in Fig. 2A, lane 7 to the signal in Fig. 2B, lane 7). This and other blots were quantified and showed that there was a significant decrease in SUMOylated PARP1 with PIAS4 KD compared to in control KD in cells treated with Talazoparib+MMS (Fig. 2C).

To investigate whether coilin could rescue the decrease in PARP1 SUMOylation when PIAS4 is reduced, control or PIAS4 KD was conducted followed by transfection with a plasmid encoding Myc-tagged coilin and then Talazoparib+MMS treatment. Ni-NTA pulldown of these lysates is shown in Fig. 2A, lane 8 and Fig. 2B, lane 8. Compared to the amount of SUMOylated PARP1 present in control or PIAS4 KD with Talazoparib+MMS treatment (Figs 2A,B, lane 7), the expression of Myc–coilin increased the amount of SUMOylated PARP1 (Figs 2A,B, compare the amount of SUMOylated PARP1 in lane 7 to that in lane 8). Quantification of the SUMOylated PARP1 with PIAS4 KD (Fig. 2D) showed that Myc–coilin increased the amount of PARP1 SUMOylation upon Talazoparib+MMS treatment (Fig. 2B, lane 8) compared to that found without ectopic coilin expression (Fig. 2B, lane 7). A representative blot showing the KD efficiency of PIAS4 is shown in Fig. S2B. Additional experimental controls were run with His–SUMO1 and Myc–coilin transfection alone (Fig. S2C). Collectively, the data presented in Figs 1 and 2 indicate that coilin might play a role, along with PIAS4, in the efficiency of PARP1 SUMOylation both in resting states and when PARP1 is recruited to sites of DNA damage.

### Coilin KD and treatment with the SUMO inhibitor TAK-981 alter PARP1 mobility

**Fig. 3.**
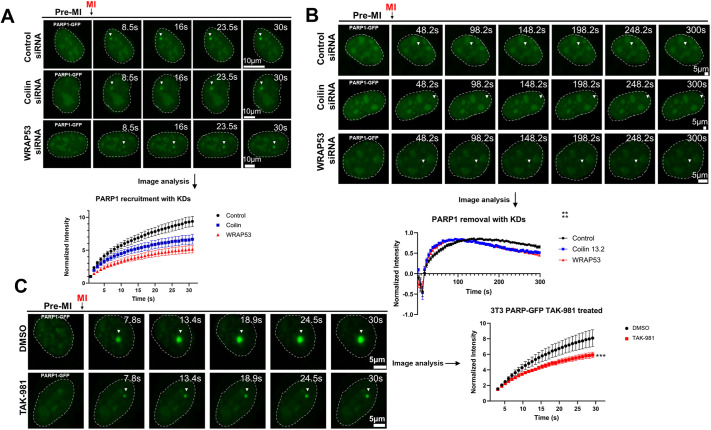
**Coilin and WRAP53 KD, as well as SUMOylation inhibition by TAK-981, alter PARP1 DDR dynamics.** (A) 3T3 cells expressing PARP1–GFP were transfected with either control, coilin or WRAP53 siRNA for 72 h. Cells were subject to Hoechst sensitization and live-cell imaging with micro-irradiation (denoted as MI) to detect and measure PARP1 recruitment to sites of DNA damage. Coilin and WRAP53 KD reduced PARP1 recruitment to sites of DNA damage (arrowheads) compared to control (**P<0.005; unpaired two-tailed Student's *t*-test). Representative images are shown with notable events indicated. An initial pre-MI acquisition image was taken and shown as labeled. The nucleus is denoted with dashed lines for each image. Scale bars (5 μm) are shown in the final image in sequence. No less than 50 cells were quantified for control and coilin siRNA treatment, and no less than 30 cells were quantified for WRAP53 siRNA treatment, obtained from four biological repeats of the experiment. (B) Cells expressing PARP1–GFP were transfected with control, coilin or WRAP53 siRNA for 72 h followed by live-cell imaging and micro-irradiation to detect PARP1 retention at sites of DNA damage. No Hoechst sensitization was conducted for this experiment. PARP1 in the coilin and WRAP53 KD conditions show less retention at the site of DNA damage compared to control. **P<0.005 (unpaired two-tailed Student's *t*-test). Representative images are shown with notable events marked. An initial pre-MI acquisition image was taken and shown as labeled. The nucleus is denoted with dashed lines for each image. Scale bars (5 μm) are shown in the final image in sequence. Quantification was done on no less than 40 cells across three biological repeats for each condition. A statistical change starts at peak to 300 s mark. (C) 3T3 cells expressing PARP1–GFP were treated with either DMSO or TAK-981 for 24 h. Cells were subject to live-cell imaging with micro-irradiation to detect and measure PARP1 recruitment to sites of DNA damage. PARP1 is less efficiently recruited to sites of DNA damage in the presence of TAK-981 compared to DMSO (vehicle). ***P<0.0005 (unpaired two-tailed Student's *t*-test). Representative images are shown with notable events marked. An initial pre-MI acquisition image was taken and shown as labeled. The nucleus is denoted with dashed lines for each image. Scale bars (5 μm) are shown in the final image in sequence. No less than 30 cells across four biologicals were quantified for both conditions. Error bars in graphs highlight mean±s.e.m.

To investigate the mechanistic implications of coilin KD on PARP1 SUMOylation, we used mouse 3T3 cells stably expressing the fusion protein PARP1–GFP to investigate the response of PARP1 to DNA damage upon reduced levels of coilin. Using live-cell microscopy, we analyzed the dynamics of PARP1 upon Hoechst sensitization and micro-irradiation in cells treated with control, coilin or WRAP53 siRNA. Coilin KD in the 3T3 line expressing PARP1–GFP is shown in Fig. S2D and E. Representative images of the response of PARP1 to DNA damage when cells were treated with either control, coilin or WRAP53 KD is shown in Fig. 3A. The results showed that cells treated with coilin and WRAP53 siRNA had significantly less PARP1 recruited to the site of DNA damage compared to cells treated with control siRNA (Fig. 3A). Because we previously saw that coilin KD leads to a decrease in SUMOylated PARP1, these results suggest the decrease in SUMOylation of PARP1 also decreases its localization to sites of DNA damage. Given that PARP1 is subsequently removed from areas of DNA damage following recruitment (Chappidi et al., 2024; Kanev et al., 2024), we next wanted to see whether these same siRNA treatments would alter PARP1 retention and removal time in these areas. In contrast to the experiment we conducted in Fig. 3A, we did not sensitize the cells with Hoechst for this analysis to facilitate quantification of the PARP1 retention time. The lack of Hoechst sensitization might account for the observed differences in the initial recruitment in Fig. 3B compared to Fig. 3A. We focused our attention for retention rate at peak recruitment (∼100 s) to the end of the experiment (300 s). We found that upon induction of double-strand DNA breaks in both coilin and WRAP53 siRNA-transfected cells, there was a higher rate of PARP1 removal from the region of interest following max recruitment. Representative images and quantification are shown in Fig. 3B. These data indicate that both coilin and WRAP53 alter PARP1 mobility to a site of DNA damage as well as the retention rate of PARP1 at these sites. Because we hypothesize that the observed influences of coilin KD on the mobility of PARP1 might be linked to the PARP1 SUMOylation status, we next looked at how treatment of 3T3 cells with TAK-981, a global SUMOylation inhibitor (Langston et al., 2021), might change PARP1 localization to sites of DNA damage. Similar to what we saw with coilin KD, cells treated with SUMOylation inhibitor TAK-981 showed a significantly lower amount of recruited PARP1 to sites of DNA damage compared to that in vehicle-treated samples. Representative images and quantification are shown in Fig. 3C. These data further enforce our hypothesis that the SUMOylation of PARP1 is facilitated by coilin, which has previously been shown to associate with PIAS4 (Sun et al., 2005), and contributes to both changes in PARP1 mobility to sites of DNA damage and retention of PARP1 at these sites.

### The SUMOylation status of coilin influences its mobility within the nucleus

**Fig. 4.**
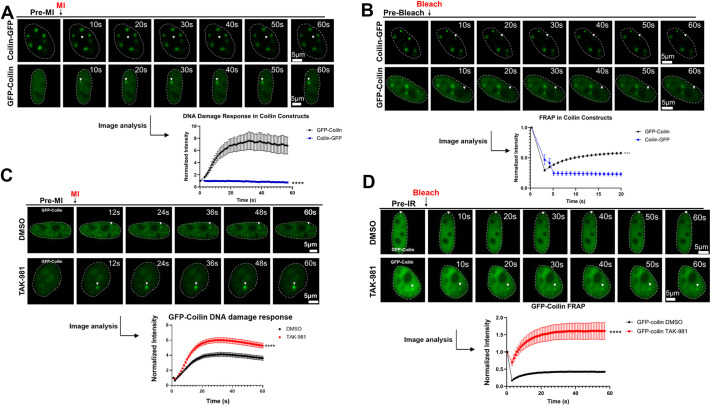
**GFP–coilin DDR dynamics and mobility are altered by TAK-981 SUMOylation inhibition.** (A) HeLa cells were transfected with either GFP–coilin or coilin–GFP for 24 h followed by live-cell imaging with micro-irradiation to detect and measure coilin recruitment to sites of DNA damage (arrowheads). Coilin–GFP showed less mobility to sites of DNA damage compared to GFP–coilin. ****P<0.00005 (unpaired two-tailed Student's *t*-test). Representative images are shown with notable events marked. An initial pre-MI acquisition image was taken and shown as labeled. The nucleus is denoted with dashed lines for each image. Scale bars (5 μm) are shown in the final image in sequence. No less than 15 cells across three biologicals were used for each construct. (B) HeLa cells were transfected with either GFP–coilin or coilin–GFP for 24 h. Cells were subject to FRAP analysis of CBs. Coilin–GFP showed less recovery of fluorescence at a CB compared to GFP–coilin. ****P<0.00005 (unpaired two-tailed Student's *t*-test). Representative images are shown with notable events marked. An initial pre-bleach acquisition image was taken and shown as labeled. The nucleus is denoted with dashed lines for each image. Scale bars (5 μm) are shown in the final image in sequence. No less than 25 cells across three biologicals were quantified for each construct. (C) HeLa cells were transfected with GFP–coilin and treated with either DMSO or TAK-981 for 24 h. Cells were subject to live-cell imaging with micro-irradiation to detect and measure coilin recruitment to sites of DNA damage. GFP–coilin was recruited more robustly to sites of DNA damage in TAK-981-treated cells compared to in DMSO-treated cells. ****P<0.00005 (unpaired two-tailed Student's *t*-test). No less than 65 cells across four biologicals were used for each treatment. An initial pre-MI acquisition image was taken and shown as labeled. The nucleus is denoted with dashed lines for each image. Scale bars (5 μm) are shown in the final image in sequence. (D) HeLa cells were transfected with GFP–coilin and treated with either DMSO or TAK-981 for 24 h followed by FRAP analysis of CBs. GFP–coilin in CBs was more mobile in cells treated with TAK-981 compared to DMSO-treated cells (****P<0.00005). An initial pre-bleach acquisition image was taken and shown as labeled. The nucleus is denoted with dashed lines for each image. Scale bars (5 μm) are shown in the final image in sequence. No less than 40 cells across three biologicals were used for each treatment. Error bars in graphs highlight mean±s.e.m.

We have published that the fusion of GFP to the N-terminus or C-terminus of coilin can influence its localization in the nucleoplasm, impact CB number and alter its SUMOylation (Hebert and Matera, 2000; Lett et al., 2023; Shpargel et al., 2003; Tucker et al., 2024). Specifically, we have shown that coilin–GFP tends to have less nucleoplasmic accumulation, generates many CB foci and is highly SUMOylated compared to GFP–coilin. Previous work has shown that GFP–coilin localizes to sites of DNA damage (Bártová et al., 2014), but no studies have evaluated whether coilin–GFP is also recruited to DNA damage sites. Given that conditions that reduce the SUMOylation of PARP1, such as coilin KD, correlate with altered PARP1 DDR dynamics, we next wanted to test whether coilin DDR dynamics are likewise influenced by its SUMOylation. To begin this investigation, we expressed GFP–coilin or coilin–GFP in HeLa cells and then evaluated the recruitment of these fusion proteins to a site of DNA damage. GFP–coilin showed a robust recruitment to the DNA damage site, in agreement with a previous report (Bártová et al., 2014) (Fig. 4A). In contrast, coilin–GFP, which is highly SUMOylated (Lett et al., 2023; Tucker et al., 2024), was not recruited to sites of DNA damage (Fig. 4A). These findings indicate the SUMOylation of coilin might reduce its mobility. As a control, we showed that GFP alone is not recruited to sites of DNA damage (Fig. S2F). We continued the investigation of coilin mobility using the two fusion proteins by conducting fluorescence recovery after photobleaching (FRAP) of CBs present in GFP–coilin- and coilin–GFP-expressing cells. Consistent with the DNA damage recruitment data, GFP–coilin in CBs showed a recovery of fluorescence whereas coilin–GFP in CBs was immobile (Fig. 4B). These data further support the hypothesis that the high level of SUMOylation found in coilin–GFP might reduce its mobility. To test this hypothesis further, cells expressing GFP–coilin were treated with vehicle or the TAK-981 SUMOylation inhibited followed by DNA damage recruitment and FRAP analysis. We saw increased recruitment of GFP–coilin to the site of DNA damage in both the rate and amount for those cells treated with TAK-981 compared to those treated with DMSO (Fig. 4C). We also observed that TAK-981 treatment increased the recovery, and, therefore, increased the mobility of GFP–coilin present in the CB compared to vehicle (Fig. 4D). These results suggest that SUMOylation is a regulator of coilin mobility, with decreased SUMOylation resulting in more mobility.

### Coilin SUMOylation and GFP–coilin mobility are influenced by the DNA-damaging agent MMS and the PARP inhibitor Talazoparib

**Fig. 5.**
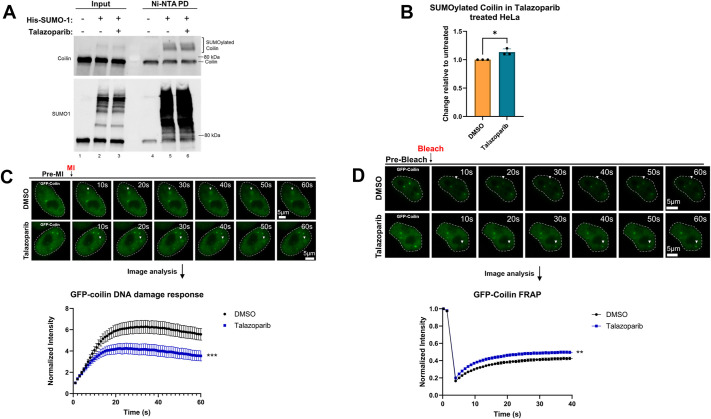
**The PARPi Talazoparib alters GFP–coilin DDR dynamics and mobility.** (A) HeLa cells were transfected with or without His–SUMO1 for 24 h and treated at 23 h post-transfection with Talazoparib or vehicle for 1 h. Lysate was subject to Ni-NTA pulldown followed by SDS-PAGE, western blotting and probing for coilin (top panel) and SUMO1 (lower panel) with the appropriate antibodies. SUMOylated coilin is indicated by the bracket. (B) A and other blots were quantified as previously described with the amount of SUMOylated coilin in DMSO treated cells set to 1. Results are mean±s.e.m., *n*=3. *P<0.05 (unpaired two-tailed Student's *t*-test). (C) HeLa cells were transfected with GFP-coilin for 24 h. At the 23 h mark, cells were treated with either DMSO or Talazoparib for 1 h. Cells were subject to live cell imaging with micro-irradiation to detect and measure coilin recruitment to sites of DNA damage. Cells treated with Talazoparib had less GFP–coilin recruitment to sites of DNA damage (arrowheads) compared to DMSO. ***P<0.0005 (unpaired two-tailed Student's *t*-test). Representative images are shown with notable events marked. An initial pre-MI acquisition image was taken and shown as labeled. The nucleus is denoted with dashed lines for each image. Scale bars (5 μm) are shown in the final image in sequence. No less than 40 cells across three biologicals were used for each treatment. (D) HeLa cells were transfected with GFP-coilin for 24 h. At the 23 h mark, cells were treated with either DMSO or Talazoparib for 1 h and then subjected to FRAP analysis of CBs. Talazoparib treatment increased the mobility of GFP–coilin in the CB compared to control. **P<0.005 (unpaired two-tailed Student's *t*-test). Representative images are shown with notable events marked. An initial pre-bleach acquisition image was taken and shown as labeled. The nucleus is denoted with dashed lines for each image. Scale bars (5 μm) are shown in the final image in sequence. No less than 50 cells across three biologicals were used for each treatment condition. Error bars in graphs in C and D highlight mean±s.e.m.

**Fig. 6.**
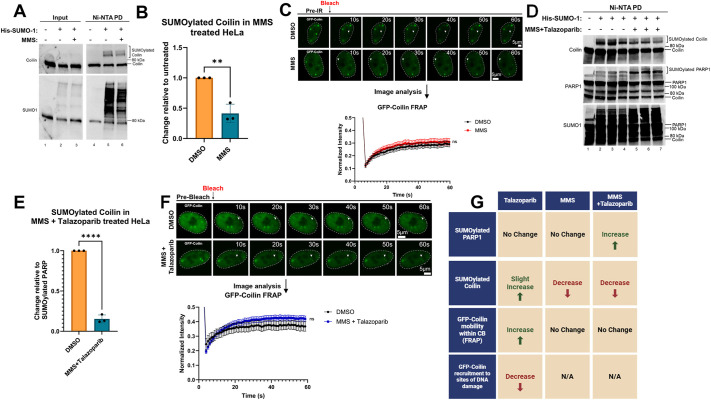
**MMS, alone or in combination with Talazoparib, differentially impacts coilin SUMOylation but does not significantly affect the mobility of GFP–coilin in CBs.** (A) HeLa cells were transfected with or without His-SUMO1 for 24 h and treated with vehicle or MMS. Lysate was subject to Ni-NTA pulldown (PD) followed by SDS-PAGE, western blotting and probing for coilin (top panel) and SUMO1 (lower panel) with the appropriate antibodies. SUMOylated coilin is indicated by the bracket. (B) A and other blots were quantified as previously described, and the amount of SUMOylated coilin was found to be decreased with MMS treatment compared to DMSO, which is set to 1. Results are mean±s.e.m., *n*=3. **P<0.005 (unpaired two-tailed Student's *t*-test). (C) HeLa cells were transfected with GFP–coilin for 24 h. At the 23 h mark, cells were treated with either DMSO or MMS for 1 h followed by FRAP analysis of CBs. No changes in the mobility of GFP–coilin were detected with MMS treatment compared to control. Representative images are shown with notable events marked. An initial pre-bleach acquisition image was taken and shown as labeled. The nucleus is denoted with dashed lines for each image. Scale bars (5 μm) are shown in the final image in sequence. No less than 30 cells across three biologicals were used for each treatment condition. (D) HeLa cells were transfected with or without His–SUMO1 for 24 h and vehicle treated or treated with MMS+Talazoparib at 23 h post-transfection for 1 h. Lysate was subject to Ni-NTA pulldown followed by SDS-PAGE, western blotting and probing for coilin (top panel), PARP1 (middle panel) and SUMO1 (lower panel) with the appropriate antibodies. SUMOylated coilin and PARP1 are indicated by a bracket. (E) D and other blots were quantified to show the change in the relative amount of SUMOylated coilin to SUMOylated PARP1in DMSO versus MMS+Talazoparib. The ratio of SUMOylated coilin/SUMOylated PARP1 with DMSO treatment was set to 1. Results are mean±s.e.m., *n*=3. ****P<0.00005 (unpaired two-tailed Student's *t*-test). (F) FRAP analysis of GFP–coilin mobility in CBs in DMSO or MMS+Talazoparib-treated cells. Representative images are shown with notable events marked. An initial pre-MI acquisition image was taken and shown as labeled. The nucleus is denoted with dashed lines for each image. Scale bars (5 μm) are shown in the final image in sequence. No less than 30 cells across three biologicals were used for each treatment condition. Arrowheads in C and F indicate the ROI. Error bars in graphs in C and F highlight mean±s.e.m. (G) Summary of the different treatments and how SUMOylated PARP1 levels, SUMOylated coilin levels and GFP–coilin mobility within the CB, and GFP–coilin recruitment to sites of DNA damage are affected by each experimental condition.

To gain a better understanding as to how coilin is involved in the DDR with PARP1, we next evaluated coilin SUMOylation and GFP–coilin mobility using treatments that increase PARP1 SUMOylation. Specifically, we treated cells with Talazoparib alone, MMS alone or a combination of MMS+Talazoparib. Lysate obtained from HeLa cells that were transfected or not with His–SUMO1 and treated with either vehicle (DMSO) or Talazoparib was subjected to Ni-NTA pulldown. Reactions were run on an SDS-PAGE followed by western blot transfer and detection of proteins using the appropriate antibodies. Higher migrating SUMOylated endogenous coilin is easily detected in the presence of His-SUMO1 (Fig. 5A, lane 5), and the amount of SUMOylated coilin is slightly increased upon treatment with Talazoparib (Fig. 5A, lane 6, quantified in Fig. 5B). In the presence of Talazoparib, GFP–coilin was recruited to micro-irradiated DNA damage sites less robustly when compared to the recruitment observed in the presence of DMSO (Fig. 5C). FRAP analysis shows that GFP-coilin mobility in the CB is slightly increased by Talazoparib compared to DMSO. We next evaluated coilin SUMOylation and GFP-coilin mobility upon treatment with MMS. The amount of SUMOylated endogenous coilin is reduced with MMS treatment compared to vehicle alone (Fig. 6A, compare lane 6 to lane 5). This and other data were quantified in Fig. 6B. Because MMS is a DNA damaging agent, the reduced level of coilin SUMOylation might be indicative of an increased fraction of mobile coilin required for the DDR. To investigate whether MMS treatment influences GFP–coilin mobility within the CB, FRAP analysis on CBs was conducted and no significant change in GFP–coilin mobility within the CB between MMS-treated and DMSO-treated cells was detected (Fig. 6C). Finally, we wanted to analyze changes in coilin SUMOylation and GFP–coilin dynamics upon treatment with MMS+Talazoparib, which traps PARP1 at the chromatin and increases its SUMOylation. Following western blot analysis of the Ni-NTA pulldown, we found significantly less SUMOylated coilin in cells treated with MMS+Talazoparib compared to that in control (Fig. 6D, lanes 2–4 compared to lanes 5–7, upper panel). PARP1 probing of the same blot showed, as expected, an increase in SUMOylated PARP1 with MMS+Talazoparib treatment (Fig. 6D, lanes 2–4 compared to lanes 5–7, middle panel). The MMS+Talazoparib treatment, therefore, decreases coilin SUMOylation but increases PARP1 SUMOylation. To better visualize the differences between coilin and PARP1 SUMOylation in the presence of MMS+Talazoparib, the amount of SUMOylated coilin relative to the amount of SUMOylated PARP1 was quantified in the vehicle-treated lanes, and this ratio was set to 1. We then quantified the amount of SUMOylated coilin to SUMOylated PARP1 upon MMS+Talazoparib. When normalized to the SUMO–coilin or SUMO–PARP1 obtained with vehicle, it could be seen that the amount of SUMOylated coilin to SUMOylated PARP1 was decreased ∼80% in the presence of MMS+Talazoparib (Fig. 6E). These findings clearly show that SUMOylation differentially regulates proteins involved in the DDR. No change in GFP–coilin mobility in the CB was detected in cells treated with MMS+Talazoparib by FRAP analysis (Fig. 6F). Data are summarized in Fig. 6G.

## DISCUSSION

Coilin is a nucleoplasmic protein that is enriched in CBs in a subset of cell types. The function of nucleoplasmic coilin is not clear, but given that coilin reduction results in a decrease of global protein SUMOylation in cell lines with or without CBs, we postulate that coilin positively contributes to the SUMOylation machinery (Lett et al., 2023). Although the exact mechanism of how coilin facilitates SUMOylation is not fully understood, it has previously been reported that coilin and PIAS4, a SUMO E3 ligase responsible for mediating SUMOylation of PARP1 (Ryu et al., 2010), associate within the nucleus and contribute to nuclear organization (Sun et al., 2005). Interestingly, like coilin, PIAS4 has been shown to localize to sites of DNA damage (Galanty et al., 2009) and is part of the DDR pathway. PARP1 has been shown to associate with coilin in both plants and *Drosophila* (Kanev et al., 2024; Kotova et al., 2009; Spechenkova et al., 2023), but we cannot detect an interaction between endogenous mammalian coilin and PARP1 by co-immunoprecipitation (co-IP) using HeLa cell lysate (our unpublished observations). Furthermore, we were unable to detect coilin–PARP1 interaction by co-IP when using overexpressed GFP-tagged PARP1 or coilin, regardless of buffer stringency conditions (our unpublished data). These findings suggest that the interaction between mammalian coilin and PARP1 is transient or disrupted by the co-IP conditions we used. We attempted to model the interaction coilin and PARP1 using AlphaFold 3 (Abramson et al., 2024). From this analysis, we found that the interface predicted template modeling score was 0.19, with any value below 0.6 showing an indication of a false prediction. Further experiments investigating the transient interaction of mammalian PARP1 and coilin, and the role that PIAS4 plays in this association, are necessary to further understand how coilin impacts the DDR. Given that PARP1, an important component of the DDR, is SUMOylated via PIAS4 and SUMOylation impacts PARP1 dynamics (Chambon et al., 1963; Krastev et al., 2022), we hypothesized that coilin contributes to the PARP1 DDR by modulating PARP1 SUMOylation. Additionally, given that coilin itself is highly SUMOylated, we further hypothesized that SUMOylation is a regulator of the coilin DDR.

### Coilin and PIAS4, a SUMO E3 ligase, positively regulate PARP1 SUMOylation

Previous work has shown that PARP1 SUMOylation is governed by PIAS4 (Krastev et al., 2022). We have found that the PIAS4-interacting protein, coilin, likewise impacts PARP1 SUMOylation (Figs 1 and 2), consistent with previous data showing the influence of coilin on the SUMOylation of other nuclear proteins (Lett et al., 2023). Given that SUMOylation occurs in groups that are functionally and physically linked (Psakhye and Jentsch, 2016), it is possible that coilin is part of a SUMOylation protein group centering on PARP1 and the DDR. Notably, treatment of cells with a combination of MMS (a DNA-damaging agent) and Talazoparib (a PARP1 inhibitor) results in PARP1 that is SUMOylated and trapped at the chromatin (Krastev et al., 2022). The reduction in the levels of coilin in the MMS+Talazoparib treatment condition results in decreased amounts of SUMOylated PARP1, demonstrating that coilin is a regulator of the PARP1 DDR. In cells with PIAS4 KD and treated with MMS+Talazoparib, ectopic expression of Myc–coilin increased PARP1 SUMOylation compared to that obtained in the absence of Myc–Coilin (Fig. 2). Collectively, these findings suggest that coilin and PIAS4 work synergistically to regulate the levels of PARP1 SUMOylation.

### PARP1 live-cell dynamics are influenced by coilin

Given that coilin promotes the SUMOylation of PARP1 in conditions (MMS+Talazoparib) that trap PARP1 at the chromatin, we next explored whether coilin alters PARP1 recruitment and retention at sites of DNA damage. Using micro-irradiation of a cell line stably expressing PARP1–GFP coupled with live cell microscopy, we found that both coilin and WRAP53 KD decrease the rate of PARP1 recruitment to sites of DNA damage (Fig. 3). Given that WRAP53 is a previously characterized component of the DDR (Bergstrand et al., 2020; Henriksson et al., 2014), the reduction of PARP1 recruitment to DNA damage sites upon WRAP53 is not necessarily surprising but nonetheless increases knowledge about the mechanisms that impact PARP1 dynamics in the DDR. In contrast, our observation that coilin KD likewise decreases PARP1 recruitment to DNA damage sites provides important new information about the function of coilin. With regards to the retention of PARP1 at DNA damage sites, which is influenced by PARP1 SUMOylation, both coilin and WRAP53 KD also result in a decrease in PARP1 retention compared to that seen upon control KD (Fig. 2). This reduction of PARP1 retention correlates with the reduced PARP1 SUMOylation observed upon coilin and WRAP53 KD (Fig. S1D). Coilin and WRAP53 are known to associate and are both enriched in CBs (Mahmoudi et al., 2010). It is therefore possible that the reduction of one of these proteins alters the interactions and functions of the other. To further implicate SUMOylation as a regulator of PARP1 dynamics, cells were treated with the SUMOylation inhibitor TAK-981. TAK-981 was shown to reduce the recruitment of PARP1 to sites of DNA damage (Fig. 3), suggesting that SUMOylated proteins contribute to the PARP1 DDR.

### GFP–coilin and coilin–GFP have different live-cell dynamics

Given that PARP1 SUMOylation is correlated with changes in live-cell dynamics as part of the DDR, we next wanted to evaluate whether the recruitment of coilin to sites of DNA damage is regulated by SUMOylation. GFP–coilin and coilin–GFP both form and localize to CBs, but are different in many aspects such as the amount of nucleoplasmic accumulation, CB number and level of SUMOylation (Hebert and Matera, 2000; Lett et al., 2023; Shpargel et al., 2003; Tucker et al., 2024). In particular, GFP–coilin is not as highly SUMOylated as coilin–GFP (Tucker et al., 2024). As GFP–coilin has been shown to be recruited to sites of UVA-induced DNA damage (Bártová et al., 2014), we evaluated the live-cell dynamics of GFP–coilin and coilin–GFP with regards to the DNA damage site recruitment. We also evaluated both constructs using FRAP of CBs. Significant differences between GFP–coilin and coilin–GFP were observed, with little recruitment of coilin–GFP to sites of DNA damage and little recovery after photobleaching of CBs formed by coilin–GFP compared to that observed with GFP–coilin (Fig. 4). These findings show that highly SUMOylated coilin-GFP is less mobile than moderately SUMOylated GFP–coilin and highlight the potential problems that the fusion of large tags may have on protein dynamics. It is important to address these problems and be aware of limitations that the N-terminal or C-terminal fusion of a GFP has on coilin interactions, functions and modifications. It has previously been seen that endogenous coilin is highly SUMOylated, similar to coilin–GFP. While GFP-coilin is also SUMOylated, coilin–GFP has been shown to better reflect the SUMOylation status of endogenous coilin (Lett et al., 2023). However, GFP–coilin presents phenotypically more similarly to endogenous coilin with a clear accumulation of nucleoplasmic coilin and a CB number similar to that observed when staining for endogenous coilin. This is in contrast to coilin–GFP, which has little nucleoplasmic signal and coilin signal localized in numerous CBs (Hebert and Matera, 2000; Shpargel et al., 2003). These differences must be considered when choosing which construct to use in a study, and should, as often as possible, be studied in tandem for the clearest insight into endogenous coilin mechanism. Given that we observed a lack of mobility of coilin–GFP (Fig. 4), we continued our studies of the dynamics of coilin in response to DNA damage using the GFP–coilin construct. We found that treatment with the SUMOylation inhibitor TAK-981 increased the recruitment of GFP–coilin to DNA damage sites and increased the recovery of fluorescence in CBs after photobleaching (Fig. 4). Together, these findings support the idea that SUMOylation decreases coilin mobility, consistent with the lack of mobility observed with highly SUMOylated coilin-GFP.

### Coilin SUMOylation and its role in the DDR

We next evaluated coilin SUMOylation and mobility using conditions (MMS+Talazoparib) that result in PARP1 SUMOylation and trapping. With regards to coilin SUMOylation, both MMS and MMS+Talazoparib treatment reduced coilin SUMOylation but Talazoparib treatment alone slightly increased coilin SUMOylation (Figs 5 and 6). Very interestingly, MMS+Talazoparib results in an inverse effect of PARP1 and coilin SUMOylation in that this treatment increases PARP1 SUMOylation but decreases coilin SUMOylation, resulting in a high ratio of SUMOylated PARP1 compared to SUMOylated coilin (Fig. 6E). DNA damage recruitment and CB FRAP analysis show that GFP–coilin mobility is slightly altered by Talazoparib (Fig. 5). FRAP analysis shows no significant alteration of GFP–coilin in CBs upon MMS and MMS+Talazoparib treatment compared to DMSO (Fig. 6). These findings demonstrate that PARP1 inhibition alone is sufficient to dysregulate GFP–coilin mobility. This work also shows that the SUMOylation of coilin is impacted by conditions that trap PARP1 in the chromatin, specifically MMS+Talazoparib. Given that coilin SUMOylation is decreased by MMS+Talazoparib, and decreased coilin SUMOylation is correlated with more coilin mobility and recruitment to sites of DNA damage, we postulate that SUMOylation is a regulator of coilin in the DDR. What specific role does coilin play in the DDR? Although far from proven, our studies show that coilin influences the dynamics of PARP1 and therefore might impact the efficient repair of double-stranded breaks. Additionally, WRAP53 also promotes the repair of double-stranded breaks upon phosphorylation of serine 64 by the ATM protein kinase (Coucoravas et al., 2017), and coilin, an interaction partner of WRAP53, might be required for the robust localization of WRAP53 to sites of DNA damage. Collectively, the studies presented here further expand the role of nucleoplasmic coilin and position coilin as a component of the DDR SUMO protein group.

## MATERIALS AND METHODS

### Cell lines, plasmids, treatments and transfections

HeLa cells were obtained from the American Type Culture Collection (ATCC) and were cultured as previously described (Enwerem et al., 2014). Mouse embryonic 3T3 cells stably expressing PARP1–GFP were obtained from the David Brown laboratory at the University of Mississippi Medical Center, MS, USA. These cells contain hygromycin selection, and were treated with fresh drug at the time of each passage. Both cell lines have been recently authenticated and tested for contamination. All siRNAs are from Integrated DNA Technologies (Coralville, IA, USA) and used with RNAiMax (Invitrogen, Carlsbad, CA, USA) according to manufacturer's protocol. Negative control, Coilin A, Coilin B, WRAP53 and Ubc9 were previously described (Lett et al., 2023; Logan et al., 2018; Poole and Hebert, 2016). The sequences of the PIAS4 siRNA used are forward (5′-CGUGCUCUACGGAAAGUACUUAAAC-3′) and reverse (5′-GUUUAAGUACUUUCCGUAGAGCACGGG-3′). All siRNA transfection was undertaken for 72 h using the RNAi Max protocol (Fisher, Pittsburg, PA, USA). All DNA transfections were 24 h using FuGene protocol (Promega, Madison, WI, USA). GFP–coilin (Hebert and Matera, 2000) and coilin–GFP plasmids (Shpargel et al., 2003) previously contained a point mutation of K496E that has been shown to impact CB formation (Basello et al., 2022). For the experiments shown here, we used constructs using WT sequences with K496. Wild-type (WT) Myc–coilin was obtained from the Lamond laboratory (University of Dundee, UK). His–SUMO1 was obtained from Addgene via the Goff laboratory (Yueh et al., 2006, Addgene #17271). Cells were treated with Talazoparib (Thermo Fisher Scientific, Waltham, MA, USA) and Talazoparib at 0.01% MMS (Thermo Fisher Scientific) and 0.1 μM Talazoparib together for 1 h. DMSO (vehicle) was added at the same volume. TAK-981 (Thermo Fisher Scientific, Waltham, MA, USA) treatment was undertaken for 24 h at a concentration of 0.1 µM. DMSO was used as the vehicle control.

### Microscopy and micro-irradiation

Mouse 3T3 cells expressing PARP1–GFP and HeLa cells were cultured as previously described (Enwerem et al., 2014) in glass bottom 35 mm MatTek dishes (Ashland, MS, USA). Cells were imaged at or ∼70% confluency with no less than 50% transfection efficiency when applicable. All cells were treated with Hoechst 62249 (Thermo Fisher Scientific) for 30 min before washing in fresh medium three times except for the PARP1 removal with KD experiment. Hoechst was added at 1 µl/ml of medium. All live-cell imaging experiments were performed in 2 ml FluoroBrite DMEM from Fisher (Pittsburg, PA, USA). Nikon C2 laser scanning confocal microscopy system was used with a 60× objective. Cells were imaged with 5% CO_2_ at 37°C. Imaging and analysis was done using Nikon NIS-elements software. Defined regions of interest (ROIs) were set as circular targets in the nucleus, outside all nucleoli. FITC filter used for focus and damaging. No cells with saturated signal were counted. For damage experiments that were Hoechst treated, 405 nm laser at power 1 was used. For damage experiments that were not Hoechst treated, 405 nm laser at power 2 was used. For FRAP experiments, 488 nm laser at power 30 was used. Scan speed for all experiments was set to 1. Time-lapse settings are specified for each experiment in the corresponding figure. Quantification was normalized for total cell bleaching and only replicates within saturation limits were counted.

### Western blotting

Cells were lysed in RIPA medium lacking EDTA and containing a Protease Inhibitor Cocktail (PIC; Thermo Fisher Scientific) as previously described (Poole and Hebert, 2016) within 5 min of retrieval from the incubator. For all Ni-NTA pulldown experiments, 20 µl/ml N-ethylmalemaleimide (Fisher) was added to RIPA to prevent de-SUMOylation. Lysate was run on a precast 7.5% Mini-Protean Gel (Bio-Rad Laboratories, Hercules, CA, USA). Western transfer and detection were conducted as previously described (Tucker et al., 2024). The primary antibodies used were anti-β-actin mouse monoclonal antibody (1:15,000; 8H10D10, Cell Signaling, Danvers, MA), anti-Myc monoclonal antibody (1:1000; 9E10, Santa Cruz Biotechnology Inc., Dallas, TX, USA), anti-SUMO-1 polyclonal antibody (1:1000; 4930, Cell Signaling, Danvers, MA), anti-coilin rabbit polyclonal antibody (1:1000; sc-32860, Santa Cruz Biotechnology Inc., Dallas, TX, USA), anti-PARP1 rabbit polyclonal antibody (1:1000; 13371, Proteintech, Rosemont, IL, USA), anti-Ubc9 rabbit monoclonal antibody (1:1000; 4918, Cell Signaling Technology, Danvers, MA, USA), and anti-PIAS4 rabbit polyclonal antibody (Proteintech, Rosemont, IL, USA). Secondary antibodies used were goat anti-mouse-IgG conjugated to HRP (1:5000; 31440, Thermo Fisher Scientific, Waltham, MA, USA) and goat anti-rabbit HRP (31460, Thermo Fisher Scientific). Where applicable, blots were sequentially probed with no stripping or deactivation of the previous secondary antibody. Bands were detected with SuperSignal West Pico Chemiluminescent Substrate (Thermo Fisher Scientific) following the manufacturer's suggested protocol. Imaging was performed on a ChemiDoc (Bio-Rad, Hercules, CA, USA). Bands were quantified using Image Lab software. Any adjustments were done to the entire gel. GraphPad Prism was used for post-hoc statistical analysis using an unpaired two-tailed Student's *t*-test and for histogram generation. Fig. S3 contains uncropped images of all western blots.

### Ni-NTA pulldown assays

Cells were rinsed in PBS and lysed in RIPA buffer minus EDTA plus PIC. Prior to lysis, 20 µl/ml of 1 M N-ethylmaleimide was added to RIPA buffer in order to prevent de-SUMOylation. Lysate was prepared as indicated in the western blotting section. Cellular lysate was then divided into 20 µl input and the rest was nutated with 40 µl 50% Ni-NTA beads (Qiagen, Germantown, MD, USA) for 1 h at 4°C. The lysate was then spun down (189 ***g*** for 1 min), supernatant removed, and the beads were washed three times with RIPA minus EDTA+PIC buffer. After the third wash, 20 µl of 2× SDS loading buffer was added to beads and the beads were boiled before running on gel for western blot analysis.

### Immunofluorescence

3T3 PARP1–GFP cells were seeded on four-well CC2 slides. Cells were fixed in 4% paraformaldehyde within 5 min of retrieval from incubator followed by permeabilization in 0.5% Triton X-100 and blocked in 10% normal goat serum (NGS) as previously described (Logan et al., 2018). Coilin was detected with 1:200 anti-coilin rabbit polyclonal antibody (H-300, Santa Cruz Biotechnology Inc., Dallas TX, USA) in 10% NGS at 37° for 30 min. Secondary antibody used was Alexa Fluor 594 goat anti-rabbit-IgG (1:200; 11012, Invitrogen). Slides were then washed in PBS and stained with DAPI to detect nuclei. Coverslip mounting was done with Antifade (Invitrogen, Carlsbad, CA, USA). Cells were imaged as previously described (Logan et al., 2018).

## Supplementary Material

10.1242/joces.263953_sup1 Supplementary information

## References

[JCS263953C1] Abramson, J., Adler, J., Dunger, J., Evans, R., Green, T., Pritzel, A., Ronneberger, O., Willmore, L., Ballard, A. J., Bambrick, J. et al. (2024). Accurate structure prediction of biomolecular interactions with AlphaFold 3. *Nature* 630, 493-500. 10.1038/s41586-024-07487-w 38718835 PMC11168924

[JCS263953C2] Andrade, L. E., Chan, E. K., Raska, I., Peebles, C. L., Roos, G. and Tan, E. M. (1991). Human autoantibody to a novel protein of the nuclear coiled body: immunological characterization and cDNA cloning of p80-coilin. *J. Exp. Med.* 173, 1407-1419. 10.1084/jem.173.6.1407 2033369 PMC2190846

[JCS263953C3] Arias Escayola, D., Zhang, C., Nischwitz, E., Schärfen, L., Dörner, K., Straube, K., Kutay, U., Butter, F. and Neugebauer, K. M. (2025). Identification of coilin interactors reveals coordinated control of Cajal body number and structure. *J. Cell Biol.* 224, e202305081. 10.1083/jcb.202305081 39602297 PMC11602656

[JCS263953C4] Bártová, E., Foltánková, V., Legartová, S., Sehnalová, P., Sorokin, D. V., Suchánková, J. and Kozubek, S. (2014). Coilin is rapidly recruited to UVA-induced DNA lesions and γ-radiation affects localized movement of Cajal bodies. *Nucleus* 5, 460-468. 10.4161/nucl.29229 24859326 PMC4133222

[JCS263953C5] Basello, D. A., Matera, A. G. and Staněk, D. (2022). A point mutation in human coilin prevents Cajal body formation. *J. Cell Sci.* 135, jcs259587. 10.1242/jcs.259587 35356988 PMC9080554

[JCS263953C6] Bergstrand, S., Böhm, S., Malmgren, H., Norberg, A., Sundin, M., Nordgren, A. and Farnebo, M. (2020). Biallelic mutations in WRAP53 result in dysfunctional telomeres, Cajal bodies and DNA repair, thereby causing Hoyeraal-Hreidarsson syndrome. *Cell Death Dis.* 11, 238. 10.1038/s41419-020-2421-4 32303682 PMC7165179

[JCS263953C7] Bryant, H. E., Schultz, N., Thomas, H. D., Parker, K. M., Flower, D., Lopez, E., Kyle, S., Meuth, M., Curtin, N. J. and Helleday, T. (2005). Specific killing of BRCA2-deficient tumours with inhibitors of poly(ADP-ribose) polymerase. *Nature* 434, 913-917. 10.1038/nature03443 15829966

[JCS263953C8] Chambon, P., Weill, J. D. and Mandel, P. (1963). Nicotinamide mononucleotide activation of new DNA-dependent polyadenylic acid synthesizing nuclear enzyme. *Biochem. Biophys. Res. Commun.* 11, 39-43. 10.1016/0006-291x(63)90024-x 14019961

[JCS263953C9] Chappidi, N., Quail, T., Doll, S., Vogel, L. T., Aleksandrov, R., Felekyan, S., Kühnemuth, R., Stoynov, S., Seidel, C. A. M., Brugués, J. et al. (2024). PARP1-DNA co-condensation drives DNA repair site assembly to prevent disjunction of broken DNA ends. *Cell* 187, 945-961.e918. 10.1016/j.cell.2024.01.015 38320550

[JCS263953C10] Coucoravas, C., Dhanjal, S., Henriksson, S., Böhm, S. and Farnebo, M. (2017). Phosphorylation of the Cajal body protein WRAP53β by ATM promotes its involvement in the DNA damage response. *RNA Biol.* 14, 804-813. 10.1080/15476286.2016.1243647 27715493 PMC5519231

[JCS263953C11] De Murcia, G. and Ménissier De Murcia, J. (1994). Poly(ADP-ribose) polymerase: a molecular nick-sensor. *Trends Biochem. Sci.* 19, 172-176. 10.1016/0968-0004(94)90280-1 8016868

[JCS263953C12] Enwerem, I. I., Velma, V., Broome, H. J., Kuna, M., Begum, R. A. and Hebert, M. D. (2014). Coilin association with Box C/D scaRNA suggests a direct role for the Cajal body marker protein in scaRNP biogenesis. *Biol. Open* 3, 240-249. 10.1242/bio.20147443 24659245 PMC3988793

[JCS263953C13] Farmer, H., McCabe, N., Lord, C. J., Tutt, A. N., Johnson, D. A., Richardson, T. B., Santarosa, M., Dillon, K. J., Hickson, I., Knights, C. et al. (2005). Targeting the DNA repair defect in BRCA mutant cells as a therapeutic strategy. *Nature* 434, 917-921. 10.1038/nature03445 15829967

[JCS263953C14] Galanty, Y., Belotserkovskaya, R., Coates, J., Polo, S., Miller, K. M. and Jackson, S. P. (2009). Mammalian SUMO E3-ligases PIAS1 and PIAS4 promote responses to DNA double-strand breaks. *Nature* 462, 935-939. 10.1038/nature08657 20016603 PMC2904806

[JCS263953C15] Gutierrez-Morton, E. and Wang, Y. (2024). The role of SUMOylation in biomolecular condensate dynamics and protein localization. *Cell Insight* 3, 100199. 10.1016/j.cellin.2024.100199 39399482 PMC11467568

[JCS263953C16] Hebert, M. D. and Matera, A. G. (2000). Self-association of coilin reveals a common theme in nuclear body localization. *Mol. Biol. Cell* 11, 4159-4171. 10.1091/mbc.11.12.4159 11102515 PMC15064

[JCS263953C17] Henriksson, S., Rassoolzadeh, H., Hedström, E., Coucoravas, C., Julner, A., Goldstein, M., Imreh, G., Zhivotovsky, B., Kastan, M. B., Helleday, T. et al. (2014). The scaffold protein WRAP53β orchestrates the ubiquitin response critical for DNA double-strand break repair. *Genes Dev.* 28, 2726-2738. 10.1101/gad.246546.114 25512560 PMC4265676

[JCS263953C18] Kanev, P. B., Varhoshkova, S., Georgieva, I., Lukarska, M., Kirova, D., Danovski, G., Stoynov, S. and Aleksandrov, R. (2024). A unified mechanism for PARP inhibitor-induced PARP1 chromatin retention at DNA damage sites in living cells. *Cell Rep.* 43, 114234. 10.1016/j.celrep.2024.114234 38758646

[JCS263953C19] Kang, M., Park, S., Park, S. H., Lee, H. G. and Park, J. H. (2022). A double-edged sword: the two faces of PARylation. *Int. J. Mol. Sci.* 23, 9826. 10.3390/ijms23179826 36077221 PMC9456079

[JCS263953C20] Kiss, T. (2004). Biogenesis of small nuclear RNPs. *J. Cell Sci.* 117, 5949-5951. 10.1242/jcs.01487 15564372

[JCS263953C21] Kotova, E., Jarnik, M. and Tulin, A. V. (2009). Poly (ADP-ribose) polymerase 1 is required for protein localization to Cajal body. *PLoS Genet.* 5, e1000387. 10.1371/journal.pgen.1000387 19229318 PMC2637609

[JCS263953C22] Krastev, D. B., Li, S., Sun, Y., Wicks, A. J., Hoslett, G., Weekes, D., Badder, L. M., Knight, E. G., Marlow, R., Pardo, M. C. et al. (2022). The ubiquitin-dependent ATPase p97 removes cytotoxic trapped PARP1 from chromatin. *Nat. Cell Biol.* 24, 62-73. 10.1038/s41556-021-00807-6 35013556 PMC8760077

[JCS263953C23] Lam, Y. W., Lyon, C. E. and Lamond, A. I. (2002). Large-scale isolation of Cajal bodies from HeLa cells. *Mol. Biol. Cell* 13, 2461-2473. 10.1091/mbc.02-03-0034 12134083 PMC117327

[JCS263953C24] Langston, S. P., Grossman, S., England, D., Afroze, R., Bence, N., Bowman, D., Bump, N., Chau, R., Chuang, B. C., Claiborne, C. et al. (2021). Discovery of TAK-981, a first-in-class inhibitor of SUMO-activating enzyme for the treatment of cancer. *J. Med. Chem.* 64, 2501-2520. 10.1021/acs.jmedchem.0c0149 33631934

[JCS263953C25] Lett, K. E., McLaurin, D. M., Tucker, S. K. and Hebert, M. D. (2023). The Cajal body marker protein coilin is SUMOylated and possesses SUMO E3 ligase-like activity. *Front. RNA Res.* 1, 1197990. 10.3389/frnar.2023.1197990 39703804 PMC11656447

[JCS263953C26] Liu, Q. and Dreyfuss, G. (1996). A novel nuclear structure containing the survival of motor neurons protein. *EMBO J.* 15, 3555-3565. 8670859 PMC451956

[JCS263953C27] Logan, M. K., Burke, M. F. and Hebert, M. D. (2018). Altered dynamics of scaRNA2 and scaRNA9 in response to stress correlates with disrupted nuclear organization. *Biol. Open* 7, bio037101. 10.1242/bio.037101 30177550 PMC6176948

[JCS263953C28] Machyna, M., Kehr, S., Straube, K., Kappei, D., Buchholz, F., Butter, F., Ule, J., Hertel, J., Stadler, P. F. and Neugebauer, K. M. (2014). The coilin interactome identifies hundreds of small noncoding RNAs that traffic through Cajal bodies. *Mol. Cell* 56, 389-399. 10.1016/j.molcel.2014.10.004 25514182

[JCS263953C29] Mahajan, R., Delphin, C., Guan, T., Gerace, L. and Melchior, F. (1997). A small ubiquitin-related polypeptide involved in targeting RanGAP1 to nuclear pore complex protein RanBP2. *Cell* 88, 97-107. 10.1016/s0092-8674(00)81862-0 9019411

[JCS263953C30] Mahmoudi, S., Henriksson, S., Weibrecht, I., Smith, S., Söderberg, O., Strömblad, S., Wiman, K. G. and Farnebo, M. (2010). WRAP53 is essential for Cajal body formation and for targeting the survival of motor neuron complex to Cajal bodies. *PLoS Biol.* 8, e1000521. 10.1371/journal.pbio.1000521 21072240 PMC2970535

[JCS263953C31] Martin, N., Schwamborn, K., Schreiber, V., Werner, A., Guillier, C., Zhang, X. D., Bischof, O., Seeler, J. S. and Dejean, A. (2009). PARP-1 transcriptional activity is regulated by sumoylation upon heat shock. *EMBO J.* 28, 3534-3548. 10.1038/emboj.2009.279 19779455 PMC2782092

[JCS263953C32] Matera, A. G. and Frey, M. R. (1998). Coiled bodies and gems: Janus or gemini? *Am. J. Hum. Genet.* 63, 317-321. 10.1086/301992 9683623 PMC1377332

[JCS263953C33] Min, A. and Im, S. A. (2020). PARP inhibitors as therapeutics: beyond modulation of PARylation. *Cancers (Basel)* 12, 394. 10.3390/cancers12020394 32046300 PMC7072193

[JCS263953C34] Minty, A., Dumont, X., Kaghad, M. and Caput, D. (2000). Covalent modification of p73alpha by SUMO-1. Two-hybrid screening with p73 identifies novel SUMO-1-interacting proteins and a SUMO-1 interaction motif. *J. Biol. Chem.* 275, 36316-36323. 10.1074/jbc.M004293200 10961991

[JCS263953C35] Morency, E., Sabra, M., Catez, F., Texier, P. and Lomonte, P. (2007). A novel cell response triggered by interphase centromere structural instability. *J. Cell Biol.* 177, 757-768. 10.1083/jcb.200612107 17548509 PMC2064277

[JCS263953C36] Moss, J. and Stanley, S. J. (1981). Amino acid-specific ADP-ribosylation. Identification of an arginine-dependent ADP-ribosyltransferase in rat liver. *J. Biol. Chem.* 256, 7830-7833. 6267027

[JCS263953C37] Pichler, A., Fatouros, C., Lee, H. and Eisenhardt, N. (2017). SUMO conjugation - a mechanistic view. *Biomol. Concepts* 8, 13-36. 10.1515/bmc-2016-0030 28284030

[JCS263953C38] Pieper, A. A., Verma, A., Zhang, J. and Snyder, S. H. (1999). Poly (ADP-ribose) polymerase, nitric oxide and cell death. *Trends Pharmacol. Sci.* 20, 171-181. 10.1016/s0165-6147(99)01292-4 10322503

[JCS263953C39] Poole, A. R. and Hebert, M. D. (2016). SMN and coilin negatively regulate dyskerin association with telomerase RNA. *Biol. Open* 5, 726-735. 10.1242/bio.018804 27215323 PMC4920197

[JCS263953C40] Psakhye, I. and Jentsch, S. (2016). Identification of Substrates of Protein-Group SUMOylation. *Methods Mol. Biol.* 1475, 219-231. 10.1007/978-1-4939-6358-4_16 27631809

[JCS263953C41] Ryu, H., Al-Ani, G., Deckert, K., Kirkpatrick, D., Gygi, S. P., Dasso, M. and Azuma, Y. (2010). PIASy mediates SUMO-2/3 conjugation of poly(ADP-ribose) polymerase 1 (PARP1) on mitotic chromosomes. *J. Biol. Chem.* 285, 14415-14423. 10.1074/jbc.M109.074583 20228053 PMC2863168

[JCS263953C42] Ryu, H., Sun, X. X., Chen, Y., Li, Y., Wang, X., Dai, R. S., Zhu, H. M., Klimek, J., David, L., Fedorov, L. M. et al. (2021). The deubiquitinase USP36 promotes snoRNP group SUMOylation and is essential for ribosome biogenesis. *EMBO Rep.* 22, e50684. 10.15252/embr.202050684 33852194 PMC8183414

[JCS263953C43] Sarangi, P. and Zhao, X. (2015). SUMO-mediated regulation of DNA damage repair and responses. *Trends Biochem. Sci.* 40, 233-242. 10.1016/j.tibs.2015.02.006 25778614 PMC4380773

[JCS263953C44] Shpargel, K. B., Ospina, J. K., Tucker, K. E., Matera, A. G. and Hebert, M. D. (2003). Control of Cajal body number is mediated by the coilin C-terminus. *J. Cell Sci.* 116, 303-312. 10.1242/jcs.00211 12482916

[JCS263953C45] Spechenkova, N., Samarskaya, V. O., Kalinina, N. O., Zavriev, S. K., MacFarlane, S., Love, A. J. and Taliansky, M. (2023). Plant poly(ADP-Ribose) polymerase 1 is a potential mediator of cross-talk between the cajal body protein coilin and salicylic acid-mediated antiviral defence. *Viruses* 15, 1282. 10.3390/v15061282 37376582 PMC10300765

[JCS263953C46] Sun, J., Xu, H., Subramony, S. H. and Hebert, M. D. (2005). Interactions between coilin and PIASy partially link Cajal bodies to PML bodies. *J. Cell Sci.* 118, 4995-5003. 10.1242/jcs.02613 16219678

[JCS263953C47] Tucker, S. K., McLaurin, D. M. and Hebert, M. D. (2024). Cajal body formation is regulated by coilin SUMOylation. *J. Cell Sci.* 137, jcs263447. 10.1242/jcs.263447 39660502 PMC11827600

[JCS263953C48] Wang, Y. and Dasso, M. (2009). SUMOylation and deSUMOylation at a glance. *J. Cell Sci.* 122, 4249-4252. 10.1242/jcs.050542 19923268 PMC2779127

[JCS263953C49] Wilkinson, K. A. and Henley, J. M. (2010). Mechanisms, regulation and consequences of protein SUMOylation. *Biochem. J.* 428, 133-145. 10.1042/bj20100158 20462400 PMC3310159

[JCS263953C50] Yang, S., Hwang, S., Kim, B., Shin, S., Kim, M. and Jeong, S. M. (2023). Fatty acid oxidation facilitates DNA double-strand break repair by promoting PARP1 acetylation. *Cell Death Dis.* 14, 435. 10.1038/s41419-023-05968-w 37454129 PMC10349888

[JCS263953C51] Young, P. J., Le, T. T., Thi Man, N., Burghes, A. H. and Morris, G. E. (2000). The relationship between SMN, the spinal muscular atrophy protein, and nuclear coiled bodies in differentiated tissues and cultured cells. *Exp. Cell Res.* 256, 365-374. 10.1006/excr.2000.4858 10772809

[JCS263953C52] Yueh, A., Leung, J., Bhattacharyya, S., Perrone, L. A., De Los Santos, K., Pu, S. Y. and Goff, S. P. (2006). Interaction of moloney murine leukemia virus capsid with Ubc9 and PIASy mediates SUMO-1 addition required early in infection. *J. Virol.* 80, 342-352. 10.1128/jvi.80.1.342-352.2006 16352559 PMC1317516

[JCS263953C53] Zhang, N., Zhang, Y., Qian, H., Wu, S., Cao, L. and Sun, Y. (2020). Selective targeting of ubiquitination and degradation of PARP1 by E3 ubiquitin ligase WWP2 regulates isoproterenol-induced cardiac remodeling. *Cell Death Differ.* 27, 2605-2619. 10.1038/s41418-020-0523-2 32139900 PMC7429876

