# Supplementary material for: Coilin and SUMOylation influence PARP1 dynamics and the DNA damage response
Source: J Cell Sci. 2025 May 21;138(10):jcs263953. doi: 10.1242/jcs.263953 (PMC12148040; doi:10.1242/jcs.263953)
Supplement: Supplementary information [file joces-138-263953-s1.pdf]

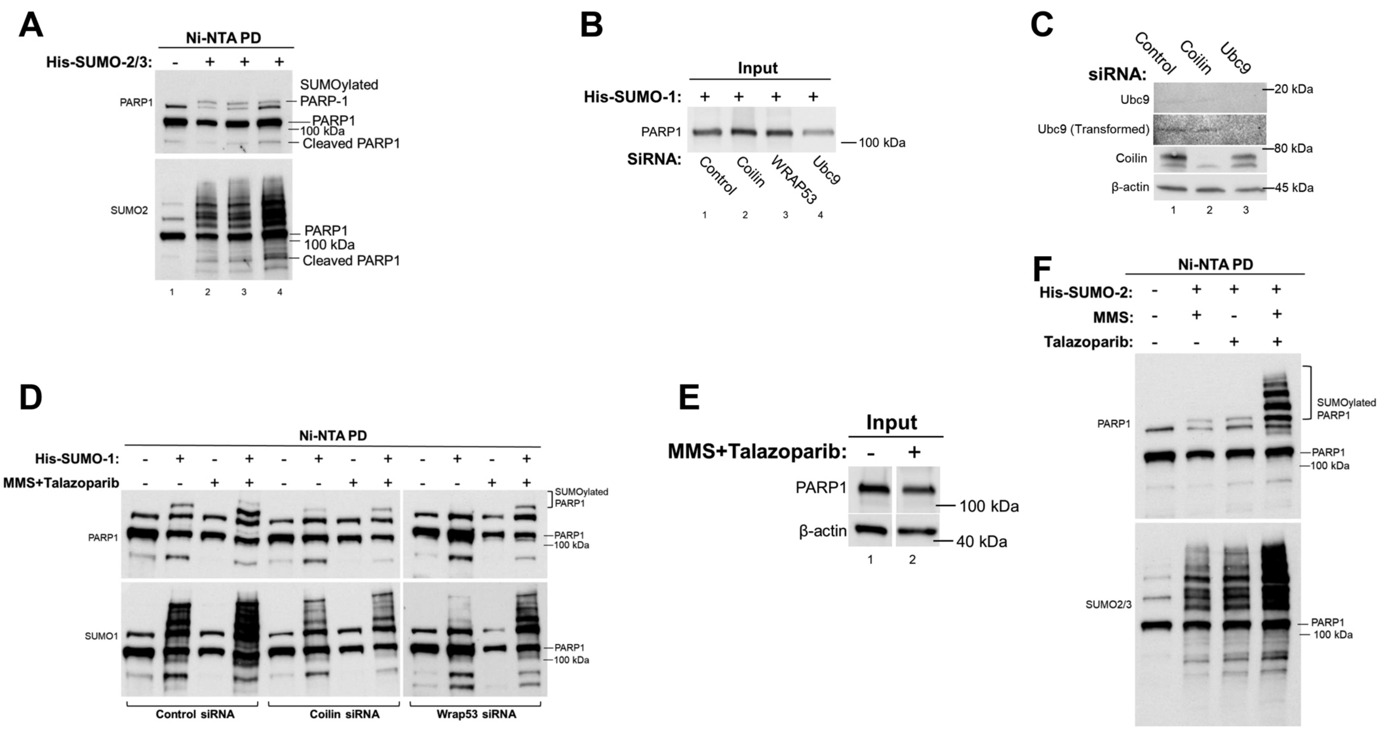

Fig. S1. A) HeLa cells untransfected or transfected with His-SUMO2 lysate was subject to Ni- NTA pulldown, SDS-PAGE, Western transfer, and probing for PARP1 (upper panel) or SUMO2/3 (lower panel). B) Representative inputs with different siRNA transfection probed for PARP1. C) Representative inputs showing typical Ubc9 and coilin KD. D) HeLa cells treated with control, coilin, or WRAP53 siRNA and then untransfected or transfected with His-SUMO1 followed by DMSO or MMS + Talazoparib treatment. Lysate was subjected to Ni-NTA pulldown, SDS-PAGE, Western transfer, and probing for PARP1 (upper panel) or SUMO1 (lower panel). Note: the control siRNA and coilin siRNA panels are the same data shown in Fig. 1F and are included for comparison with WRAP53 siRNA. E) Representative inputs of HeLa cells DMSO or MMS + Talazoparib treatment and probed for PARP1. F) HeLa cells untransfected or transfected with His-SUMO2 and untreated or treated with MMS, Talazoparib, or a combination of both. Lysate was subject to Ni-NTA pulldown, SDS PAGE, Western transfer, and probing for PARP1 (upper panel) and SUMO2/3 (lower panel).

Journal of Cell Science • Supplementary information

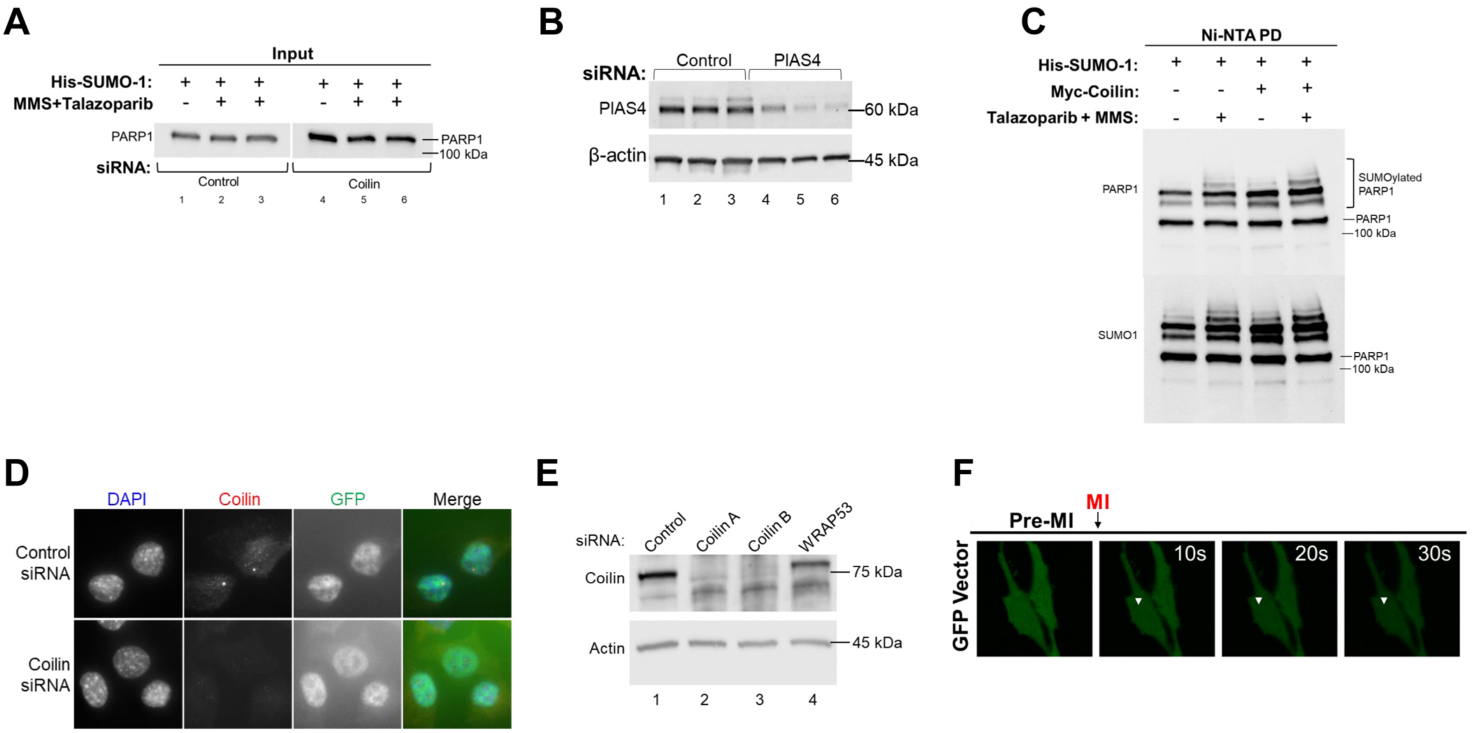

Fig. S2. A) PARP1 probing of representative inputs of HeLa cells transfected with control or coilin siRNA and then transfected with His-SUMO1 followed by DMSO or MMS + Talazoparib treatment. B) Representative KD of PIAS4 in HeLa cells. C) HeLa cells were transfected with His-SUMO1 alone or co transfected with myc-coilin and untreated or treated with MMS + Talazoparib. Lysate was subject to Ni-NTA pulldown, SDS PAGE, Western transfer, and probing for PARP1 (upper panel) and SUMO1 (lower panel). D) Immunofluorescence analysis of the 3T3 cell line expressing PARP1-GFP with coilin siRNA treatment for 72 hours. Anti-coilin signal in red, PARP1-GFP is green and DAPI (blue) stains the nucleus. E) Representative knockdown of coilin in the 3T3 cell line expressing PARP1-GFP transfected with control, coilin A, coilin B, and WRAP53 siRNA. F) HeLa cells transfected with GFP vector and subject to live cell imaging with micro-irradiation to detect recruitment to sites of DNA damage, serving as negative control.

Journal of Cell Science • Supplementary information

Figure 1 Full Westerns

Figure 1A:

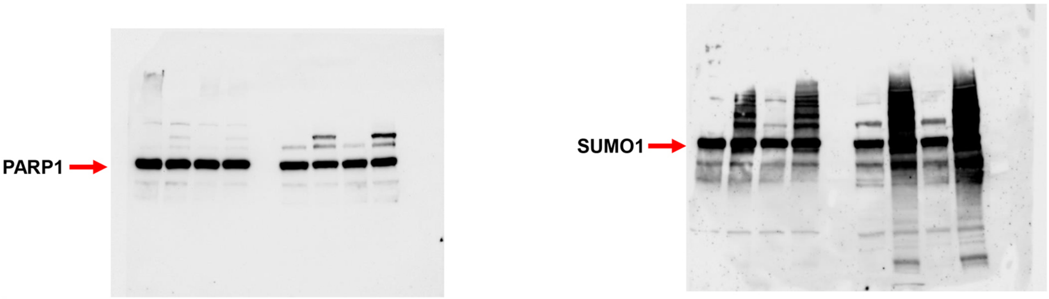

Figure 1B:

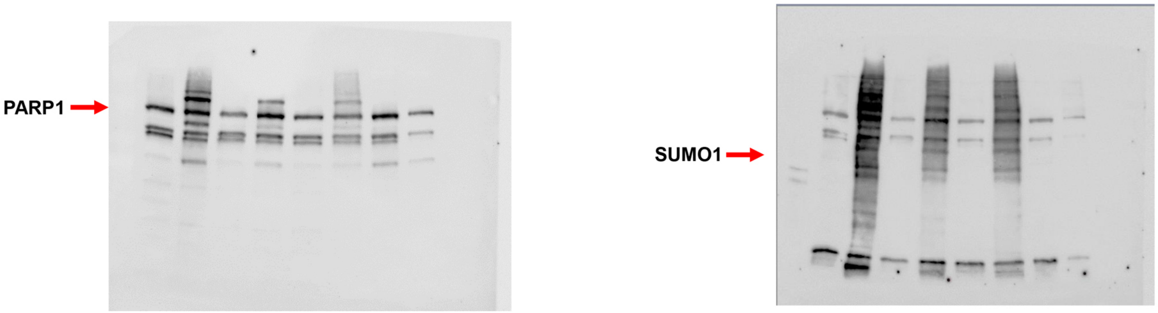

Figure 1 Full Westerns cont’d

Figure 1D:

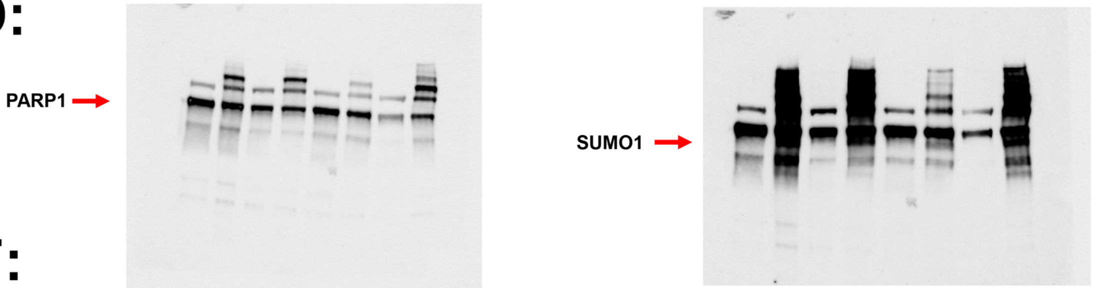

Figure 1F:

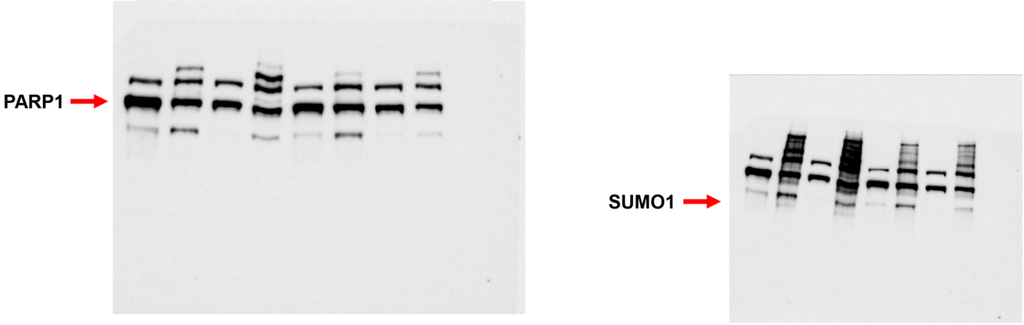

Journal of Cell Science • Supplementary information

Figure 2 Full Westerns

Figure 2A:

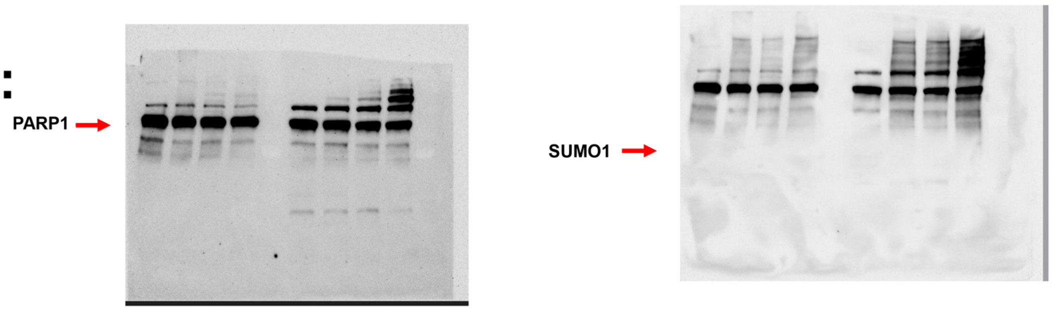

Figure 2B:

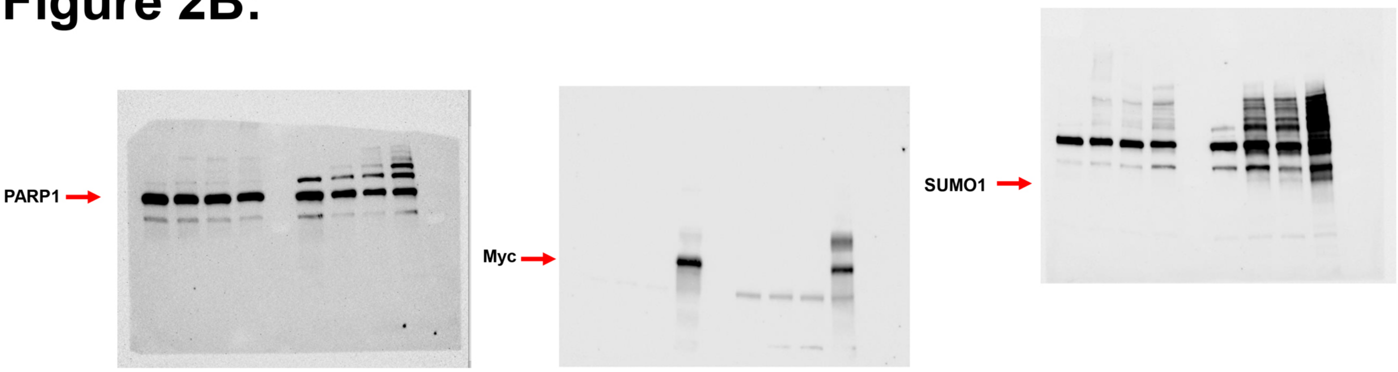

Figure 5 Full Westerns

Figure 5A:

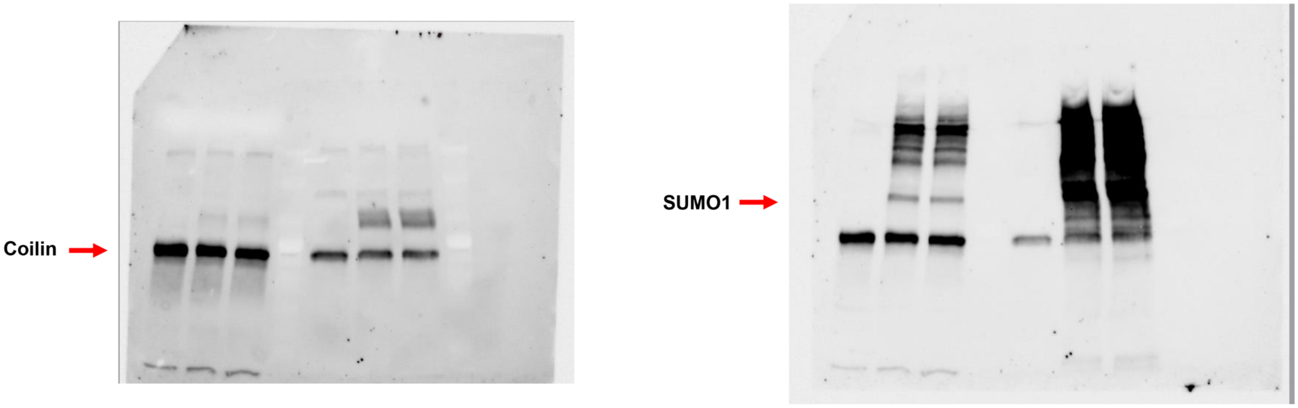

Journal of Cell Science • Supplementary information

Figure 6 Full Westerns

Figure 6A:

Coilin

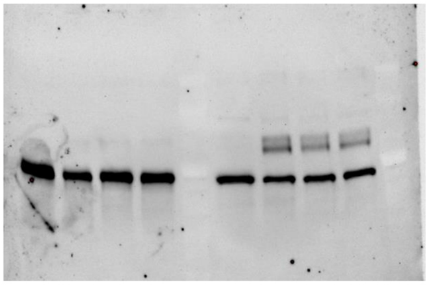

SUMO1

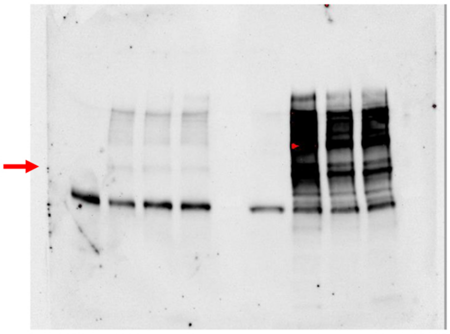

Figure 6D:

Coilin

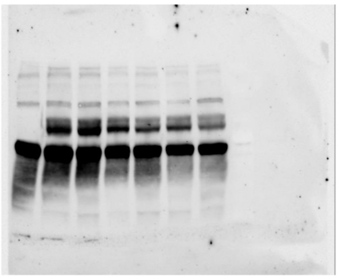

PARP1

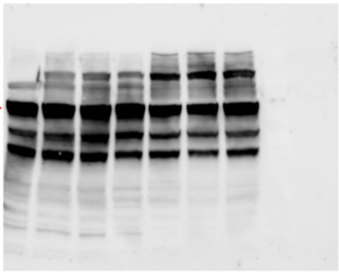

SUMO1

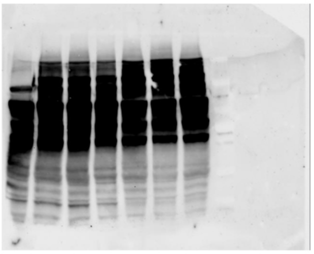

Supplemental Figure 1 Full Westerns

Supplemental Figure 1A:

PARP1

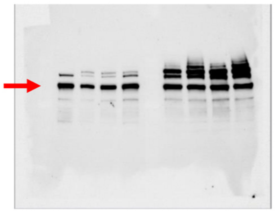

SUMO 2/3

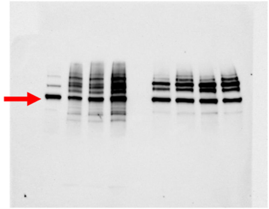

Supplemental Figure 1B:

PARP1

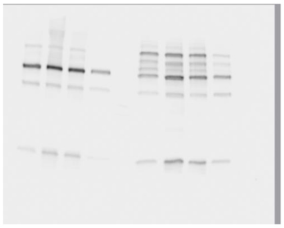

Supplemental Figure 1C:

Ubc9

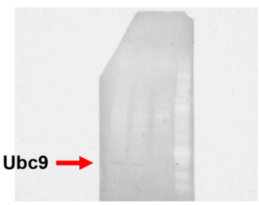

Ubc9

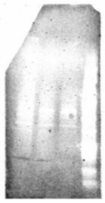

Coilin

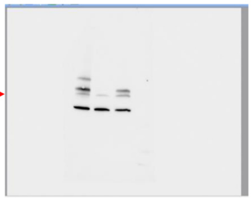

β-actin

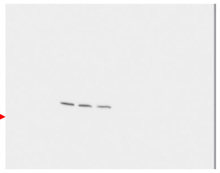

Journal of Cell Science • Supplementary information

Supplemental Figure 1 Full Westerns cont’d

Supplemental Figure 1D:

PARP1
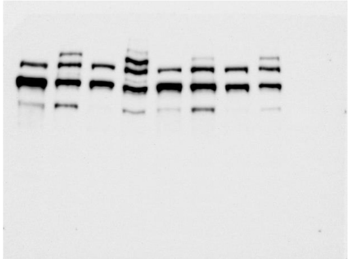

SUMO1
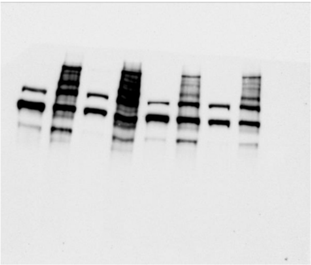

PARP1
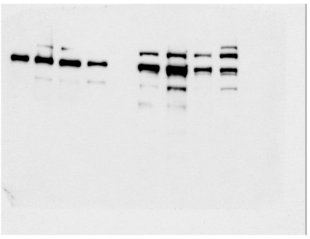

SUMO1
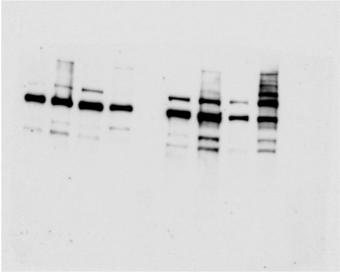

Supplemental Figure 1E:

PARP1
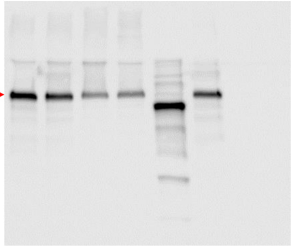

β-actin
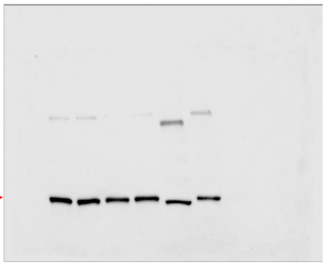

Supplemental Figure 1 Full Westerns cont’d

Supplemental Figure 1F:

PARP1
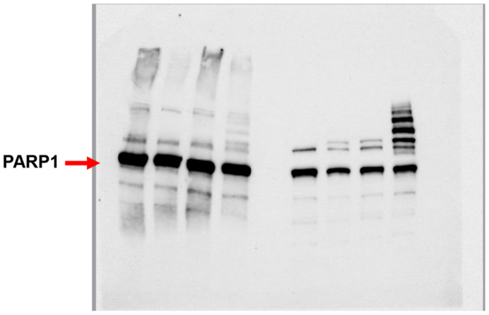

SUMO 2/3
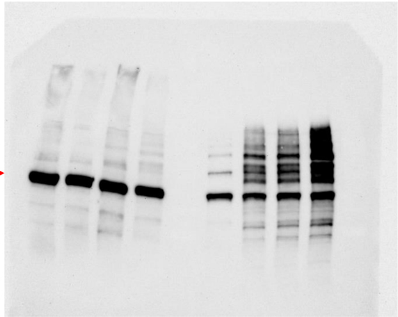

Supplemental Figure 2 Full Westerns

Supplemental Figure 2A:

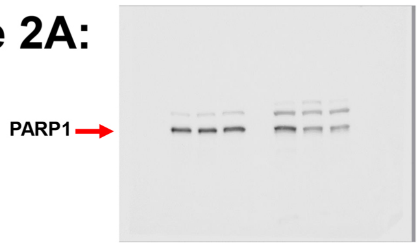

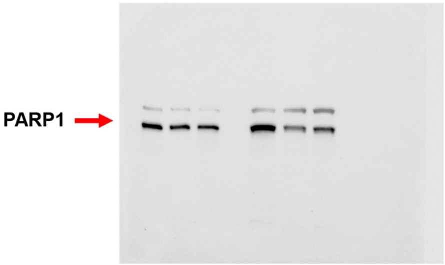

Supplemental Figure 2B:

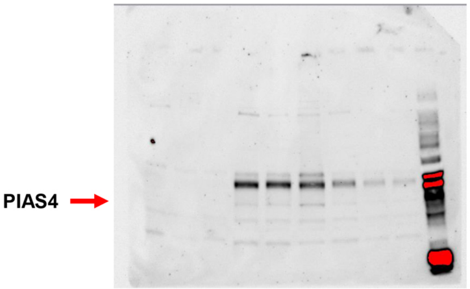

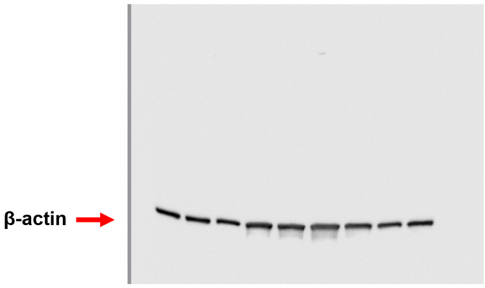

Supplemental Figure 2 Full Westerns cont'd

Supplemental Figure 2C:

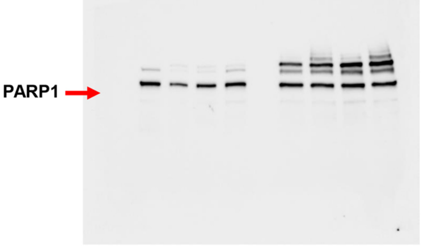

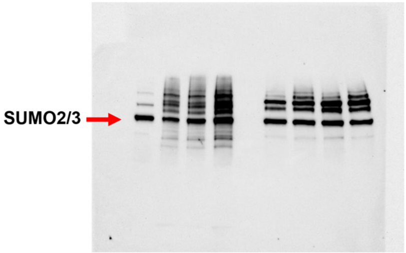

Supplemental Figure 2E:

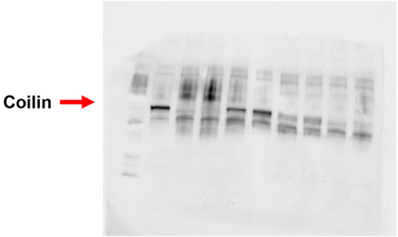

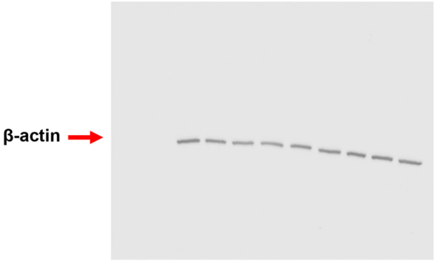

Fig. S3. Blot transparency figure showing uncropped images of all Western blots.

Journal of Cell Science • Supplementary information
